# Enhancing cognitive performance prediction by white matter hyperintensity connectivity assessment

**DOI:** 10.1093/brain/awae315

**Published:** 2024-10-14

**Authors:** Marvin Petersen, Mirthe Coenen, Charles DeCarli, Alberto De Luca, Ewoud van der Lelij, Alzheimer’s Disease Neuroimaging Initiative, Michael Weiner, Paul Aisen, Ronald Petersen, Michael Weiner, Paul Aisen, Ronald Petersen, Clifford R Jack, William Jagust, Susan Landau, Monica Rivera-Mindt, Ozioma Okonkwo, Leslie M Shaw, Edward B Lee, Arthur W Toga, Laurel Beckett, Danielle Harvey, Robert C Green, Andrew J Saykin, Kwangsik Nho, Richard J Perrin, Duygu Tosun, Pallavi Sachdev, Robert C Green, Erin Drake, Tom Montine, Cat Conti, Michael W Weiner, Rachel Nosheny, Diana Truran Sacrey, Juliet Fockler, Melanie J Miller, Catherine Conti, Winnie Kwang, Chengshi Jin, Adam Diaz, Miriam Ashford, Derek Flenniken, Ronald Petersen, Paul Aisen, Michael Rafii, Rema Raman, Gustavo Jimenez, Michael Donohue, Jennifer Salazar, Andrea Fidell, Virginia Boatwright, Justin Robison, Caileigh Zimmerman, Yuliana Cabrera, Sarah Walter, Taylor Clanton, Elizabeth Shaffer, Caitlin Webb, Lindsey Hergesheimer, Stephanie Smith, Sheila Ogwang, Olusegun Adegoke, Payam Mahboubi, Jeremy Pizzola, Cecily Jenkins, Laurel Beckett, Danielle Harvey, Michael Donohue, Naomi Saito, Adam Diaz, Kedir Adem Hussen, Ozioma Okonkwo, Monica Rivera-Mindt, Hannatu Amaza, Mai Seng Thao, Shaniya Parkins, Omobolanle Ayo, Matt Glittenberg, Isabella Hoang, Kaori Kubo Germano, Joe Strong, Trinity Weisensel, Fabiola Magana, Lisa Thomas, Vanessa Guzman, Adeyinka Ajayi, Joseph Di Benedetto, Sandra Talavera, Clifford R Jack, Joel Felmlee, Nick C Fox, Paul Thompson, Charles DeCarli, Arvin Forghanian-Arani, Bret Borowski, Calvin Reyes, Caitie Hedberg, Chad Ward, Christopher Schwarz, Denise Reyes, Jeff Gunter, John Moore-Weiss, Kejal Kantarci, Leonard Matoush, Matthew Senjem, Prashanthi Vemuri, Robert Reid, Ian Malone, Sophia I Thomopoulos, Talia M Nir, Neda Jahanshad, Alexander Knaack, Evan Fletcher, Danielle Harvey, Duygu Tosun-Turgut, Stephanie Rossi Chen, Mark Choe, Karen Crawford, Paul A Yushkevich, Sandhitsu Das, William Jagust, Susan Landau, Robert A Koeppe, Gil Rabinovici, Victor Villemagne, Brian LoPresti, Richard J Perrin, John Morris, Erin Franklin, Haley Bernhardt, Nigel J Cairns, Lisa Taylor-Reinwald, Leslie Shaw, Edward B Lee, M Y Virginia Lee, Magdalena Korecka, Magdalena Brylska, Yang Wan, J Q Trojanowki, Arthur W Toga, Karen Crawford, Scott Neu, Andrew J Saykin, Kwangsik Nho, Tatiana M Foroud, Taeho Jo, Shannon L Risacher, Hannah Craft, Liana G Apostolova, Kelly Nudelman, Kelley Faber, Zoë Potter, Kaci Lacy, Rima Kaddurah-Daouk, Li Shen, Jason Karlawish, Claire Erickson, Joshua Grill, Emily Largent, Kristin Harkins, Michael W Weiner, Leon Thal, Zaven Kachaturian, Richard Frank, Peter J Snyder, Neil Buckholtz, John K Hsiao, Laurie Ryan, Susan Molchan, Zaven Khachaturian, Maria Carrillo, William Potter, Lisa Barnes, Marie Bernard, Hector González, Carole Ho, John K Hsiao, Jonathan Jackson, Eliezer Masliah, Donna Masterman, Ozioma Okonkwo, Richard Perrin, Laurie Ryan, Nina Silverberg, Lisa Silbert, Jeffrey Kaye, Sylvia White, Aimee Pierce, Amy Thomas, Tera Clay, Daniel Schwartz, Gillian Devereux, Janet Taylor, Jennifer Ryan, Mike Nguyen, Madison DeCapo, Yanan Shang, Lon Schneider, Cynthia Munoz, Diana Ferman, Carlota Conant, Katherin Martin, Kristin Oleary, Sonia Pawluczyk, Elizabeth Trejo, Karen Dagerman, Liberty Teodoro, Mauricio Becerra, Madiha Fairooz, Sonia Garrison, Julia Boudreau, Yair Avila, James Brewer, Aaron Jacobson, Antonio Gama, Chi Kim, Emily Little, Jennifer Frascino, Nichol Ferng, Socorro Trujillo, Judith Heidebrink, Robert Koeppe, Steven MacDonald, Dariya Malyarenko, Jaimie Ziolkowski, James O'Connor, Nicole Robert, Suzan Lowe, Virginia Rogers, Ronald Petersen, Barbara Hackenmiller, Bradley Boeve, Colleen Albers, Connie Kreuger, David Jones, David Knopman, Hugo Botha, Jessica Magnuson, Jonathan Graff-Radford, Kerry CrawleyW, Michael Schumacher, Sanna McKinzie, Steven Smith, Tascha Helland, Val Lowe, Vijay Ramanan, Valory Pavlik, Jacob Faircloth, Jeffrey Bishop, Jessica Nath, Maria ChaudharyP, Maria Kataki, Melissa Yu, Nathiel Pacini, Randall Barker, Regan Brooks, Ruchi Aggarwal, Lawrence Honig, Yaakov Stern, Akiva Mintz, Jonathan Cordona, Michelle Hernandez, Justin Long, Abbey Arnold, Alex Groves, Anna Middleton, Blake Vogler, Cierra McCurry, Connie Mayo, Cyrus Raji, Fatima S Amtashar, Heather KlempW, Heather Nicole Elmore, James Ruszkiewicz, Jasmina Kusuran, Jasmine Stewart, Jennifer Horenkamp, Julia Greeson, Kara Wever, Katie Vo, Kelly Larkin, Lesley Rao, Lisa Schoolcraft, Lora Gallagher, Madeline Paczynski, Maureen McMillan, Michael Holt, Nicole Gagliano, Rachel Henson, Renee LaBarge, Robert Swarm, Sarah MunieN, Serena Cepeda, Stacey WintertonN, Stephen Hegedus, TaNisha Wilson, Tanya Harte, Zach Bonacorsi, David Geldmacher, Amber Watkins, Brandi BargerRT, Bryan Smelser, Charna Bates, Cynthia Stover, Emily McKinley, Gregory Ikner, Haley Hendrix, Harold Matthew Cooper, Jennifer Mahaffey, Lindsey Booth Robbins, Loren Brown Ashley, Marissa Natelson-Love, Princess Carter, Veronika Solomon, Hillel Grossman, Alexandra Groome, Allison Ardolino, Anthony Kaplan, Faye Sheppard, Genesis Burgos-Rivera, Gina Garcia-Camilo, Joanne Lim, Judith Neugroschl, Kimberly Jackson, Kirsten Evans, Laili Soleimani, Mary Sano, Nasrin Ghesani, Sarah Binder, Xiomara Mendoza Apuango, Ajay Sood, Amelia Troutman, Kimberly Blanchard, Arlene Richards, Grace Nelson, Kirsten HendricksonN, Erin Yurko, Jamie Plenge, Victoria Rufo, Raj Shah, Ranjan Duara, Brendan Lynch, Cesar Chirinos, Christine Dittrich, Debbie Campbell, Diego Mejia, Gilberto Perez, Helena Colvee, Joanna Gonzalez, Josalen Gondrez, Joshua Knaack, Mara Acevedo, Maria Cereijo, Maria Greig-Custo, Michelle Villar, Morris Wishnia, Sheryl Detling, Warren Barker, Marilyn Albert, Abhay Moghekar, Barbara Rodzon, Corey Demsky, Gregory Pontone, Jim Pekar, Leonie Farrington, Martin Pomper, Nicole Johnson, Tolulope Alo, Martin Sadowski, Anaztasia Ulysse, Arjun Masurkar, Brittany Marti, David Mossa, Emilie Geesey, Emily Petrocca, Evan Schulze, Jennifer Wong, Joseph Boonsiri, Sunnie Kenowsky, Tatianne Martinez, Veronica Briglall, P Doraiswamy Murali, Adaora Nwosu, Alisa Adhikari, Cammie Hellegers, Jeffrey Petrella, Olga James, Terence Wong, Thomas Hawk, Sanjeev Vaishnavi, Hannah McCoubrey, Ilya Nasrallah, Rachel Rovere, Jeffrey Maneval, Elizabeth Robinson, Francisco Rivera, Jade Uffelman, Martha Combs, Patricia O'Donnell, Sara Manning, Richard King, Alayne NietoN, Amanda Glueck, Anjana Mandal, Audrie Swain, Bethanie Gamble, M R Beverly Meacham, Denece Forenback, Dorothy Ross, Elizabeth Cheatham, Ellen Hartman, Gary Cornell, Jordan Harp, Laura Ashe, Laura Goins, Linda Watts, Morgan Yazell, Prabin Mandal, Regan BucklerN, Sylvia Vincent, Triana Rudd, Oscar Lopez, Ann Malia Arlene, Caitlin Chiado, Cary Zik, James Ruszkiewicz, Kathleen Savage, Linda Fenice, MaryAnn Oakley, Paige C Tacey, Sarah Berman, Sarah Bowser, Stephen Hegedus, Xanthia Saganis, Anton Porsteinsson, Abigail Mathewson, Asa Widman, Bridget Holvey, Emily Clark, Esmeralda Morales, C Iris Young, James Ruszkiewicz, Kevin Hopkins, Kimberly Martin, Nancy Kowalski, Rebecca Hunt, Roberta Calzavara, Russell Kurvach, C Stephen D'Ambrosio, Gaby Thai, Beatriz Vides, Brigit Lieb, Catherine McAdams-Ortiz, Cyndy Toso, Ivan Mares, Kathryn Moorlach, Luter Liu, Maria Corona, Mary Nguyen, Melanie Tallakson, Michelle McDonnell, Milagros Rangel, Neetha Basheer, Patricia Place, Romina Romero, Steven Tam, Trung Nguyen, Abey Thomas, Alexander (Alex) Frolov, Alka Khera, Amy Browning, Brendan Kelley, R Courtney Dawson, Dana Mathews, Elaine Most, Elizeva Phillips, Lynn Nguyen, Maribel Nunez, Matalin Miller, R Jones Matthew, Natalie Martinez, C Rebecca Logan, Roderick McColl, Sari Pham, Tiffani Fox, Tracey Moore, Allan Levey, Abby Brown, Andrea Kippels, Ashton Ellison, Casie Lyons, Chadwick Hales, Cindy Parry, Courtney Williams, Elizabeth McCorkle, Guy Harris, Heather Rose, Inara Jooma, Jahmila Al-Amin, James Lah, James Webster, Jessica Swiniarski, Latasha Chapman, Laura Donnelly, Lauren Mariotti, Mary Locke, Phyllis Vaughn, Rachael Penn, Sallie Carpentier, Samira Yeboah, Sarah Basadre, Sarah Malakauskas, Stefka Lyron, Tara Villinger, Terra Burney, Jeffrey Burns, C Ala Abusalim, Alexandra Dahlgren, Alexandria Montero, Anne Arthur, Heather Dooly, Katelynn Kreszyn, Katherine Berner, Lindsey Gillen, Maria Scanlan, Mercedes Madison, Nicole Mathis, Phyllis Switzer, Ryan Townley, Samantha Fikru, Samantha Sullivan, Ella Wright, Maryam Beigi, Anthony Daley, Ashley Ko, Brittney Luong, Glen Nyborg, Jessica Morales, Kelly Durbin, Lauren Garcia, Leila Parand, Lorena Macias, Lorena Monserratt, Maya Farchi, Pauline Wu, Robert Hernandez, Thao Rodriguez, Neill Graff-Radford, A'llana Marolt, Anton Thomas, Deborah Aloszka, Ercilia Moncayo, Erin Westerhold, Gregory Day, Kandise Chrestensen, Mary Imhansiemhonehi, Sanna McKinzie, Sochenda Stephens, Sylvia Grant, Jared Brosch, Amy Perkins, Aubree Saunders, Debra Silberberg Kovac, Heather Polson, Isabell Mwaura, Kassandra Mejia, Katherine Britt, Kathy King, Kayla Nichols, Kayley Lawrence, Lisa Rankin, Martin Farlow, Patricia Wiesenauer, Robert Bryant, Scott Herring, Sheryl Lynch, Skylar Wilson, Traci Day, William Korst, Christopher van Dyck, Adam Mecca, Alyssa Miller, Amanda Brennan, Amber Khan, Audrey Ruan, Carol Gunnoud, Chelsea Mendonca, Danielle Raynes-Goldfinger, Elaheh Salardini, Elisa Hidalgo, Emma Cooper, Erawadi Singh, Erin Murphy, Jeanine May, Jesse Stanhope, Jessica Lam, Julia Waszak, Kimberly Nelsen, Kimberly Sacaza, Mayer Joshua Hasbani, Meghan Donahue, Ming-Kai Chen, Nicole Barcelos, Paul Eigenberger, Robin Bonomi, Ryan O'Dell, Sarah Jefferson, Siddharth Khasnavis, Stephen Smilowitz, Susan DeStefano, Susan Good, Terry Camarro, Vanessa Clayton, Yanis Cavrel, YuQuan “Oliver” Lu, Howard Chertkow, Howard Bergman, Chris Hosein, Sandra Black, Anish Kapadia, Aparna Bhan, Benjamin Lam, Christopher Scott, Gillian Gabriel, Jennifer Bray, Ljubica Zotovic, Maria Samira Gutierrez, Mario Masellis, Marjan Farshadi, Maurylette Gui, Meghan Mitchellc, Rebecca Taylor, Ruby Endre, Zhala Taghi-Zada, Robin Hsiung, Carolyn English, Ellen Kim, Eugene Yau, Haley Tong, Laura Barlow, Lauren Jennings, Michele Assaly, Paula Nunes, Tahlee Marian, Andrew Kertesz, John Rogers, Dick Trost, Dylan Wint, Charles Bernick, Donna Munic, Ian Grant, Aaliyah Korkoyah, Ali Raja, Allison Lapins, Caila Ryan, Jelena Pejic, Kailey Basham, Leena Lukose, Loreece Haddad, Lucas Quinlan, Nathaniel Houghtaling, Carl Sadowsky, Walter Martinez, Teresa Villena, Brigid Reynolds, Angelica Forero, Carolyn Ward, Emma Brennan, Esteban Figueroa, Giuseppe Esposito, Jessica Mallory, Kathleen Johnson, Kathryn Turner, Katie Seidenberg, Kelly McCann, Margaret Bassett, Melanie Chadwick, Raymond Scott Turner, Robin Bean, Saurabh Sharma, Gad Marshall, Aferdita Haviari, C Alison Pietras, Bradley Wallace, Catherine Munro, Gladiliz Rivera-Delpin, Hadley Hustead, Isabella Levesque, Jennifer Ramirez, M R Karen Nolan, Kirsten Glennon, Mariana Palou, Michael Erkkinen, Nicole DaSilva, Pamela Friedman, Regina M Silver, Ricardo Salazar, Roxxanne Polleys, Scott McGinnis, Seth Gale, Tia Hall, Tuan Luu, Steven Chao, Emmeline Lin, Jaila Coleman, Kevin Epperson, Minal Vasanawala, Alireza Atri, Amy Rangel, Brittani Evans, Candy Monarrez, Carol Cline, Carolyn Liebsack, Daniel Bandy, Danielle Goldfarb, Debbie Intorcia, Jennifer Olgin, Kelly Clark, Kelsey King, Kylee York, Marina Reade, Michael Callan, Michael Glass, Michaela Johnson, Michele Gutierrez, Molly Goddard, Nadira Trncic, Parichita Choudhury, Priscilla Reyes, Serena Lowery, Shaundra Hall, Sonia Olgin, Stephanie de Santiago, Michael Alosco, Alyssa Ton, Amanda Jimenez, Andrew Ellison, Anh Tran, Brandon Anderson, Della Carter, Donna Veronelli, Steven Lenio, Eric Steinberg, Jesse Mez, Jason Weller, Jennifer Johns, Jesse Mez, Jessica Harkins, Alexa Puleio, Ina Hoti, Jane Mwicigi, Alexa Puleio, Michael Alosco, Olivia Schultz, Mona Lauture, Eric Steinberg, Ridiane Denis, Ronald Killiany, Sarab Singh, Steven Lenio, Wendy Qiu, Ycar Devis, Thomas Obisesan, Andrew Stone, Debra Ordor, Ifreke Udodong, Immaculata Okonkwo, Javed Khan, Jillian Turner, Kyliah Hughes, Oshoze Kadiri, Charles Duffy, Ariana Moss, Katherine Stapleton, Maria Toth, Marianne Sanders, Martin Ayres, Melissa Hamski, Parianne Fatica, Paula Ogrocki, Sarah Ash, Stacy Pot, Doris Chen, Andres Soto, Costin Tanase, David Bissig, Hafsanoor Vanya, Heather Russell, Hitesh Patel, Hongzheng Zhang, Kelly Wallace, Kristi Ayers, Maria Gallegos, Martha Forloines, Meghan Sinn, Queennie Majorie S Kahulugan, Richard Isip, Sandra Calderon, Talia Hamm, Michael Borrie, T-Y Lee, Rob Bartha, Sterling Johnson, Sanjay Asthana, Cynthia M Carlsson, Allison Perrin, Pierre Tariot, Adam Fleisher, Stephanie Reeder, Horacio Capote, Allison Emborsky, Anna Mattle, Bela Ajtai, C Benjamin Wagner, Bennett Myers, Daryn Slazyk, C Delaney Fragale, Erin Fransen, Heather Macnamara, C Jonathan Falletta, Joseph Hirtreiter, Laszlo Mechtler, Megan King, Michael Asbach, Michelle Rainka, Richard Zawislak, Scott Wisniewski, C Stephanie O'Malley, Tatiana Jimenez-Knight, Todd Peehler, Traci Aladeen, Vernice Bates, Violet Wenner, Wisam Elmalik, Douglas W Scharre, Arun Ramamurthy, Soumya Bouchachi, Maria Kataki, Rawan Tarawneh, Brendan Kelley, Dzintra Celmins, Alicia Leader, Chris Figueroa, Heather Bauerle, Katlynn Patterson, Michael Reposa, Steven Presto, Tuba Ahmed, Wendy Stewart, Godfrey D Pearlson, Karen Blank, Karen Anderson, Robert B Santulli, Eben S Schwartz, Jeff Williamson, Alicia Jessup, Andrea Williams, Crystal Duncan, Abigail O'Connell, Karen Gagnon, Ezequiel Zamora, James Bateman, Freda Crawford, Deb Thompson, Eboni Walker, Jennifer Rowell, Mikell White, Phillip “Hunter” Ledford, Sarah Bohlman, Susan Henkle, Joseph Bottoms, Lena Moretz, Bevan Hoover, Michael Shannon, C Samantha Rogers, Wendy Baker, William Harrison, Chuang-Kuo Wu, Alexis DeMarco, Ava Stipanovich, Daniel Arcuri, Jan Clark, Jennifer Davis, Kerstin Doyon, Marie Amoyaw, Mauro Veras Acosta, R Ronald Bailey, Scott Warren, Terry Fogerty, Victoria Sanborn, Meghan Riddle, Stephen Salloway, Paul Malloy, Stephen Correia, Charles Windon, Morgan Blackburn, Howard J Rosen, Bruce L Miller, Amanda Smith, Ijeoma Mba, Jenny Echevarria, Juris Janavs, Emily Roglaski, Meagan Yong, Rebecca Devine, Hamid Okhravi, Edgardo Rivera, Teresa Kalowsky, Caroline Smith, Christina Rosario, Joseph Masdeu, Richard Le, Maushami Gurung, Marwan Sabbagh, Angelica Garcia, Micah Ellis Slaughter, Nadeen Elayan, Skieff Acothley, Nunzio Pomara, Raymundo Hernando, Vita Pomara, Chelsea Reichert, Olga Brawman-Mintzer, Allison Acree, Arthur Williams, Campbell Long, Rebecca Long, Paul Newhouse, Sydni Jenee Hill, Amy Boegel, Sudha Seshadri, Amy Saklad, Floyd Jones, William Hu, V Sotelo, Yaneicy Gonazalez Rojas, Jacobo Mintzer, Crystal Flynn Longmire, Kenneth Spicer, Frederik Barkhof, Thomas Benke, Christopher P L H Chen, Peter Dal-Bianco, Anna Dewenter, Marco Duering, Christian Enzinger, Michael Ewers, Lieza G Exalto, Evan M Fletcher, Nicolai Franzmeier, Saima Hilal, Edith Hofer, Huiberdina L Koek, Andrea B Maier, Pauline M Maillard, Cheryl R McCreary, Janne M Papma, Yolande A L Pijnenburg, Reinhold Schmidt, Eric E Smith, Rebecca M E Steketee, Esther van den Berg, Wiesje M van der Flier, Vikram Venkatraghavan, Narayanaswamy Venketasubramanian, Meike W Vernooij, Frank J Wolters, Xin Xu, Andreas Horn, Kaustubh R Patil, Simon B Eickhoff, Götz Thomalla, J Matthijs Biesbroek, Geert Jan Biessels, Bastian Cheng

**Affiliations:** Department of Neurology, University Medical Center Hamburg-Eppendorf, Hamburg 20251Germany; Department of Neurology and Neurosurgery, University Medical Center Utrecht Brain Center, Utrecht 3584 CX, The Netherlands; Department of Neurology, University of California, Davis, CA 95616USA; Department of Neurology and Neurosurgery, University Medical Center Utrecht Brain Center, Utrecht 3584 CX, The Netherlands; Division Imaging and Oncology, Image Sciences Institute, UMC Utrecht, Utrecht 3584 CX, The Netherlands; Department of Neurology and Neurosurgery, University Medical Center Utrecht Brain Center, Utrecht 3584 CX, The Netherlands; Department of Radiology and Nuclear Medicine, Amsterdam UMC, Vrije Universiteit Amsterdam, Amsterdam 1081 BT, The Netherlands; Queen Square Institute of Neurology and Centre for Medical Image Computing, University College, London WC1N 3BG, UK; Clinic of Neurology, Medical University Innsbruck, Innsbruck 6020, Austria; Department of Pharmacology, Yong Loo Lin School of Medicine, National University of Singapore, Singapore 119228, Singapore; Department of Psychological Medicine, Yong Loo Lin School of Medicine, National University of Singapore, Singapore 119228, Singapore; Memory, Aging and Cognition Center, National University Health System, Singapore 119228, Singapore; Department of Neurology, Medical University Vienna, Vienna 1090, Austria; Institute for Stroke and Dementia Research (ISD), LMU University Hospital, LMU Munich, Munich 81377, Germany; Institute for Stroke and Dementia Research (ISD), LMU University Hospital, LMU Munich, Munich 81377, Germany; Medical Image Analysis Center (MIAC) and Department of Biomedical Engineering, University of Basel, Basel 4051, Switzerland; Division of General Neurology, Department of Neurology, Medical University Graz, Graz 8036, Austria; Division of Neuroradiology, Interventional and Vascular Radiology, Department of Radiology, Medical University of Graz, Graz 8036, Austria; Institute for Stroke and Dementia Research (ISD), LMU University Hospital, LMU Munich, Munich 81377, Germany; Department of Neurology and Neurosurgery, University Medical Center Utrecht Brain Center, Utrecht 3584 CX, The Netherlands; Department of Neurology, University of California, Davis, CA 95616USA; Institute for Stroke and Dementia Research (ISD), LMU University Hospital, LMU Munich, Munich 81377, Germany; Department of Psychological Medicine, Yong Loo Lin School of Medicine, National University of Singapore, Singapore 119228, Singapore; Saw Swee Hock School of Public Health, National University of Singapore and National University Health System, Singapore 119228, Singapore; Division of Neurogeriatrics, Department of Neurology, Medical University of Graz, Graz 8036, Austria; Institute for Medical Informatics, Statistics and Documentation, Medical University of Graz, Graz 8036, Austria; Department of Neurology and Neurosurgery, University Medical Center Utrecht Brain Center, Utrecht 3584 CX, The Netherlands; Department of Geriatric Medicine, University Medical Center Utrecht, Utrecht University, Utrecht 3584 CX, The Netherlands; Department of Pharmacology, Yong Loo Lin School of Medicine, National University of Singapore, Singapore 119228, Singapore; Department of Psychological Medicine, Yong Loo Lin School of Medicine, National University of Singapore, Singapore 119228, Singapore; Memory, Aging and Cognition Center, National University Health System, Singapore 119228, Singapore; Department of Neurology, University of California, Davis, CA 95616USA; Department of Clinical Neurosciences and Radiology and Hotchkiss Brain Institute, University of Calgary, Calgary AB T2N 4N1, Canada; Alzheimer Center Erasmus MC, Erasmus MC University Medical Center, Rotterdam 3015 GD, The Netherlands; Department of Neurology, Erasmus MC University Medical Center, Rotterdam 3015 GD, The Netherlands; Department of Internal Medicine, Erasmus MC University Medical Center, Rotterdam 3015 GD, The Netherlands; Alzheimer Center Amsterdam, Department of Neurology, Amsterdam Neuroscience, Amsterdam UMC, Vrije Universiteit Amsterdam, Amsterdam 1081 BT, The Netherlands; Division of Neurogeriatrics, Department of Neurology, Medical University of Graz, Graz 8036, Austria; Institute for Medical Informatics, Statistics and Documentation, Medical University of Graz, Graz 8036, Austria; Department of Clinical Neurosciences and Radiology and Hotchkiss Brain Institute, University of Calgary, Calgary AB T2N 4N1, Canada; Alzheimer Center Amsterdam, Department of Neurology, Amsterdam Neuroscience, Amsterdam UMC, Vrije Universiteit Amsterdam, Amsterdam 1081 BT, The Netherlands; Alzheimer Center Erasmus MC, Erasmus MC University Medical Center, Rotterdam 3015 GD, The Netherlands; Department of Neurology, Erasmus MC University Medical Center, Rotterdam 3015 GD, The Netherlands; Alzheimer Center Amsterdam, Department of Neurology, Amsterdam Neuroscience, Amsterdam UMC, Vrije Universiteit Amsterdam, Amsterdam 1081 BT, The Netherlands; Alzheimer Center Amsterdam, Department of Neurology, Amsterdam Neuroscience, Amsterdam UMC, Vrije Universiteit Amsterdam, Amsterdam 1081 BT, The Netherlands; Memory, Aging and Cognition Center, National University Health System, Singapore 119228, Singapore; Raffles Neuroscience Center, Raffles Hospital, Singapore 119228, Singapore; Alzheimer Center Erasmus MC, Erasmus MC University Medical Center, Rotterdam 3015 GD, The Netherlands; Department of Radiology and Nuclear Medicine, Erasmus MC University Medical Center, Rotterdam 3015 GD, The Netherlands; Department of Epidemiology, Erasmus MC University Medical Center, Rotterdam 3015 GD, The Netherlands; Department of Radiology and Nuclear Medicine, Erasmus MC University Medical Center, Rotterdam 3015 GD, The Netherlands; Department of Epidemiology, Erasmus MC University Medical Center, Rotterdam 3015 GD, The Netherlands; Memory, Aging and Cognition Center, National University Health System, Singapore 119228, Singapore; School of Public Health and the Second Affiliated Hospital of School of Medicine, Zhejiang University, Zhejiang 310009, China; Department of Neurology with Experimental Neurology, Charité - Universitätsmedizin Berlin, Movement Disorders and Neuromodulation Unit, Berlin 10117, Germany; Department of Neurology, Psychiatry, and Radiology, Center for Brain Circuit Therapeutics, Brigham and Women’s Hospital, Harvard Medical School, Boston, MA 02115, USA; Institute for Systems Neuroscience, Medical Faculty, Heinrich-Heine University Düsseldorf, Düsseldorf 40225, Germany; Institute of Neuroscience and Medicine, Brain and Behaviour (INM-7), Research Center Jülich, Jülich 52428, Germany; Institute for Systems Neuroscience, Medical Faculty, Heinrich-Heine University Düsseldorf, Düsseldorf 40225, Germany; Institute of Neuroscience and Medicine, Brain and Behaviour (INM-7), Research Center Jülich, Jülich 52428, Germany; Department of Neurology, University Medical Center Hamburg-Eppendorf, Hamburg 20251Germany; Department of Neurology and Neurosurgery, University Medical Center Utrecht Brain Center, Utrecht 3584 CX, The Netherlands; Department of Neurology, Diakonessenhuis Hospital, Utrecht 3582 KE, The Netherlands; Department of Neurology and Neurosurgery, University Medical Center Utrecht Brain Center, Utrecht 3584 CX, The Netherlands; Department of Neurology, University Medical Center Hamburg-Eppendorf, Hamburg 20251Germany

**Keywords:** vascular cognitive impairment, white matter hyperintensities, dementia, lesion network mapping, magnetic resonance imaging, cerebral small vessel disease

## Abstract

White matter hyperintensities of presumed vascular origin (WMH) are associated with cognitive impairment and are a key imaging marker in evaluating brain health. However, WMH volume alone does not fully account for the extent of cognitive deficits and the mechanisms linking WMH to these deficits remain unclear. Lesion network mapping (LNM) enables us to infer if brain networks are connected to lesions and could be a promising technique for enhancing our understanding of the role of WMH in cognitive disorders. Our study employed LNM to test the following hypotheses: (i) LNM-informed markers surpass WMH volumes in predicting cognitive performance; and (ii) WMH contributing to cognitive impairment map to specific brain networks.

We analysed cross-sectional data of 3485 patients from 10 memory clinic cohorts within the Meta VCI Map Consortium, using harmonized test results in four cognitive domains and WMH segmentations. WMH segmentations were registered to a standard space and mapped onto existing normative structural and functional brain connectome data. We employed LNM to quantify WMH connectivity to 480 atlas-based grey and white matter regions of interest (ROI), resulting in ROI-level structural and functional LNM scores. We compared the capacity of total and regional WMH volumes and LNM scores in predicting cognitive function using ridge regression models in a nested cross-validation. LNM scores predicted performance in three cognitive domains (attention/executive function, information processing speed, and verbal memory) significantly better than WMH volumes. LNM scores did not improve prediction for language functions. ROI-level analysis revealed that higher LNM scores, representing greater connectivity to WMH, in grey and white matter regions of the dorsal and ventral attention networks were associated with lower cognitive performance.

Measures of WMH-related brain network connectivity significantly improve the prediction of current cognitive performance in memory clinic patients compared to WMH volume as a traditional imaging marker of cerebrovascular disease. This highlights the crucial role of network integrity, particularly in attention-related brain regions, improving our understanding of vascular contributions to cognitive impairment. Moving forward, refining WMH information with connectivity data could contribute to patient-tailored therapeutic interventions and facilitate the identification of subgroups at risk of cognitive disorders.

## Teaser

Petersen *et al.* show that assessing the brain circuitry affected by cerebrovascular white matter lesions improves the accuracy with which cognitive performance can be predicted in memory clinic patients compared to using lesion volume alone. This method could help identify individuals at risk of cognitive disorders.

**See O'Sullivan (https://doi.org/10.1093/brain/awae377) for a scientific commentary on this article.**

## Introduction

Cerebral small vessel disease (CSVD) is a major driver of vascular cognitive impairment (VCI) and often also contributes to dementia with a primary neurodegenerative or mixed pathology.^1^ White matter hyperintensities (WMH) are the signature imaging marker of CSVD, and mark sites of white matter disintegration caused by microangiopathic axonal loss and demyelination.^2,3^ However, a comprehensive understanding of mechanisms linking WMH to their broad range of clinical manifestations, specifically cognitive impairment, is still lacking.

Although there is a well documented association between WMH volumes and cognitive functions at the group level, the association between WMH volume and symptom severity demonstrates considerable variability with some individuals exhibiting fewer symptoms despite high WMH burden and vice versa.^4^ The apparent complexity of this relationship underscores the need for improved techniques for disease quantification to more accurately predict individual cognitive impairment for effective diagnostics and ultimately targeted treatment of VCI patients.^5^ An important part of the variation in the impact of WMH on cognition may be explained by loco-regional WMH effects. For example, lesion-symptom inference techniques have linked cognitive impairment to WMH located in strategic white matter regions, independent of total WMH volume.^4,6-8^

However, these findings might not fully reflect the complexity of CSVD-related cognitive impairment, which is thought to emerge from disturbances in the interplay of large-scale brain networks involving cortical and subcortical grey matter areas, interconnected by white matter tracts.^9,10^ In recent years, advanced imaging analysis models have been developed to comprehensively capture lesion effects on brain circuitry.^11^ Specifically, lesion network mapping (LNM) techniques capitalize on advanced neuroimaging to map lesions on reconstructions of the human brain network.^12^ By that, a lesion’s connectivity to different brain regions can be quantified—i.e. the lesion’s network embedding is measured—allowing one to infer which regions are disconnected.^11,13^ LNM measures have been associated with clinical symptoms in a variety of neurological disorders that can be understood as ‘disconnection syndromes’, such as stroke or multiple sclerosis.^13-15^

Here, we propose LNM as a technique to quantify WMH connectivity for improved prediction of cognitive performance in VCI. We employ LNM on a large scale, multicentre dataset, integrating cognitive test results and MRI-based WMH segmentations from 3485 patients of 10 memory clinic cohorts through the Meta VCI Map Consortium.^6,16^ Our hypotheses are twofold: (i) LNM-based measures of WMH connectivity surpass WMH volumes in predicting cognitive performance; and (ii) WMH contributing to cognitive deficits map to specific brain networks that functionally determine their symptom profile.

## Materials and methods

### Study population

**Figure 1:**
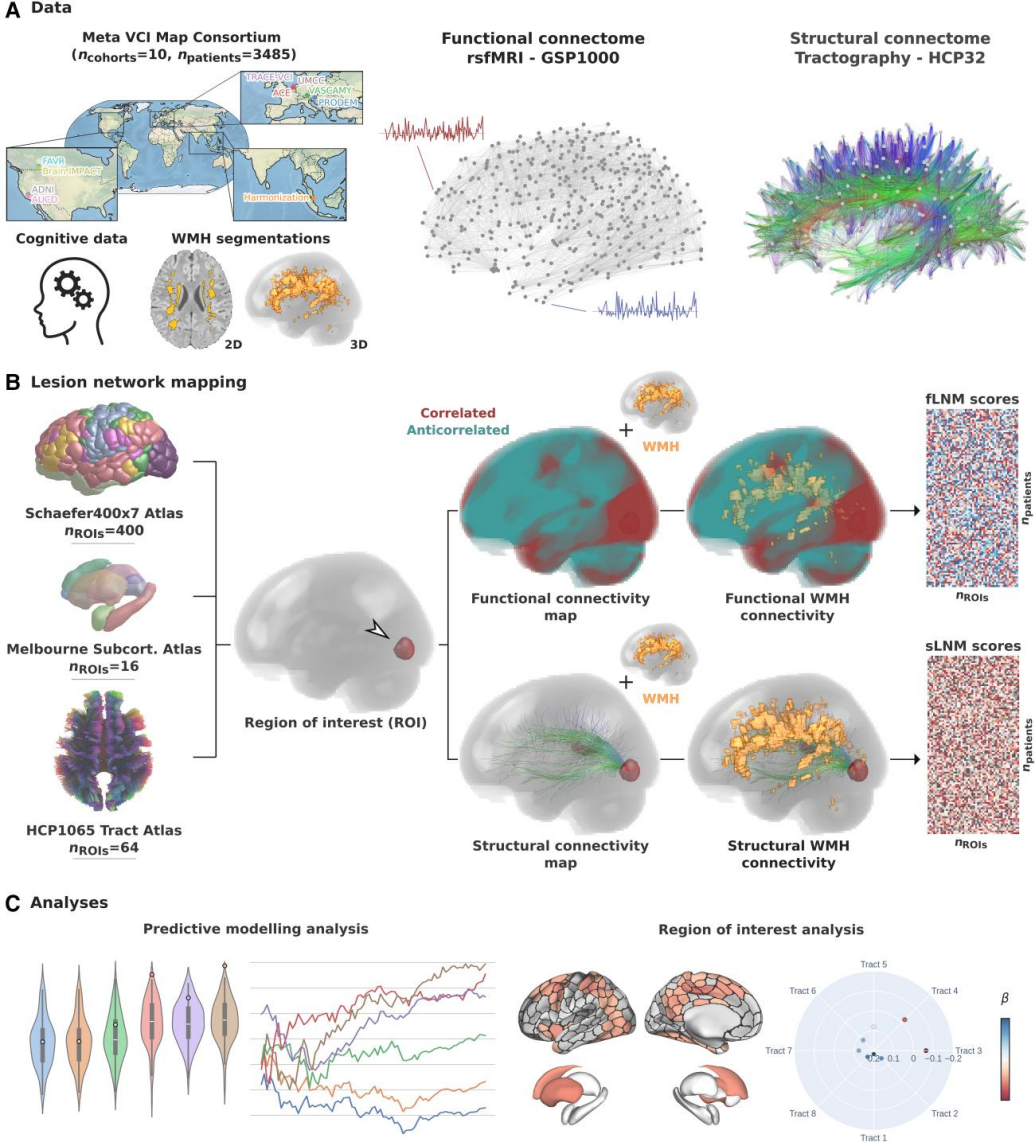
**Methodology.** (**A**) Data from 10 memory clinic cohorts of the Meta VCI Map Consortium were used including harmonized cognitive scores and WMH segmentations in MNI space. For functional lesion network mapping (fLNM) we employed the GSP1000 normative functional connectome comprising resting state fMRI (rsfMRI) data from 1000 healthy GSP participants. For structural lesion network mapping (sLNM), we used the HCP32 normative structural connectome based on diffusion-weighted imaging data from 32 healthy HCP participants, detailing the fibre bundle architecture. (**B**) LNM was performed to quantify the functional and structural connectivity of white matter hyperintensities of presumed vascular origin (WMH) to multiple regions of interest (ROIs) (Schaefer400 × 7 cortical, Melbourne Subcortical Atlas subcortical, HCP1065 white matter areas). For this, voxel-level functional and structural connectivity maps were computed for each ROI, reflecting resting state blood oxygen level-dependent (BOLD) correlations or anatomical connection strength via tractography streamlines, respectively. ROIwise LNM scores were derived by averaging voxel-level connectivity indices within the normalized WMH masks, considering only positive correlation coefficients for functional mapping. This resulted in a matrix for both fLNM and sLNM scores per ROI per patient (*n*_ROIs_ × *n*_patients_). The matrices shown in the figure are populated with random data only serving as a visual aid. (**C**) The fLNM and sLNM scores across patients were used in predictive models to estimate cognitive domain scores (predictive modelling analysis) and analysed in permutation-based general linear models to identify regions significantly influencing the cognitive domain-WMH disconnectivity relationship at the ROI level (ROI-level inferential statistics). GSP = Genomic Superstruct Project; HCP = Human Connectome Project.

Methodological details are illustrated in Fig. 1. We examined previously harmonized, cross-sectional clinical and imaging data of 3485 patients from 10 memory clinic cohorts of the Meta VCI Map Consortium.^6,16^ Meta VCI map is a multi-site collaboration for conducting meta-analyses of strategic lesion topography in vascular cognitive impairment. The memory clinic cohorts included in this study comprise the Alzheimer Center Erasmus MC (ACE, *n* = 52, The Netherlands), Alzheimer’s Disease Neuroimaging Initiative (ADNI, *n* = 994, USA),^17^ UC Davis Alzheimer’s Disease Center Diversity Cohort (AUCD, *n* = 641, USA),^18^ BrainIMPACT (*n* = 53, Canada),^19^ Functional Assessment of Vascular Reactivity (FAVR, *n* = 47, Canada),^19^ Harmonization (*n* = 207, Singapore),^4^ Prospective Dementia Registry (PRODEM, *n* = 367, Austria),^20^ TRACE-VCI (*n* = 821, The Netherlands),^21^ Utrecht Memory Clinic Cohort (UMCC, *n* = 227, The Netherlands) and VASCAMY (*n* = 76, Germany). All cohorts include patients assessed at outpatient memory clinics for cognitive symptoms, undergoing structural MRI alongside neuropsychological tests of cognitive performance. Patients with cognitive impairment due to non-vascular, non-neurodegenerative causes (e.g. excessive alcohol use disorder, cerebral malignancies, multiple sclerosis) or monogenic disorders (e.g. CADASIL) were excluded. Further details on each cohort, including sample-specific inclusion and exclusion criteria were reported previously.^6^

### Ethics approval

All cohorts received the requisite ethical and institutional approval in accordance with local regulations, which included informed consent, to allow data acquisition and sharing.^6^

### Cognitive assessments

Detailed harmonization procedures, including specific test-to-domain assignments, were reported previously.^22^ Neuropsychological tests from participating cohorts were norm-referenced against local norms or a healthy control group, and adjusted on the individual subject level for age, sex and education. These tests were categorized into four cognitive domains: attention/executive function, information processing speed, language and verbal memory. Within these domains, norm-referenced neuropsychological test scores were *z*-scored and averaged to obtain cognitive domain scores (*z*-scores), which capture individual-level cognitive domain performance relative to healthy controls.

### White matter hyperintensity segmentation

WMH segmentations were provided by the participating centres or performed at the UMC Utrecht (ACE cohort). Segmentation masks were obtained applying established automated neuroimaging software on fluid-attenuated inversion recovery (FLAIR) MRI.^23^ WMH segmentations were spatially normalized to the Montreal Neurological Institute (MNI)-152 template.^24^ To ensure registration quality, the normalized WMH masks were visually inspected and patients with failed registrations were excluded. Furthermore, random subsamples of normalized WMH segmentations were returned to the respective participating institutions to confirm the data quality. WMH segmentation masks were used to compute the total WMH volume as well as tract-level WMH volumes for each of the 64 white matter fibre tracts of the HCP1065 Tract Atlas.^25^ Details on cohort-specific segmentation and registration procedures were reported previously.^6,26^

### Lesion network mapping

LNM was performed to quantify the functional and structural connectivity of WMH to cortical, subcortical and white matter regions of interest (ROIs) following previous procedures.^13,15,27,28^ ROIs were defined in MNI space using the following established atlases for comprehensive brain coverage: Schaefer400 × 7 Atlas (*n*_ROIs_ = 400), the Melbourne Subcortical Atlas (*n*_ROIs_ = 16) and the HCP1065 Tract Atlas (*n*_ROIs_ = 64) (Fig. 1B).^25,29,30^ For visualization of the investigated HCP1065 tracts, see Supplementary Fig. 1.

Functional lesion network mapping (fLNM) was conducted using a normative functional connectome, derived from resting state functional MRI (fMRI) scans of 1000 healthy individuals from the Genomic Superstruct Project (GSP1000).^31,32^ Preprocessing was performed using a modified version of the Computational Brain Imaging Group (CBIG) fMRI preprocessing pipeline (https://github.com/bchcohenlab/CBIG/tree/master/stable_projects/preprocessing/CBIG_fMRI_Preproc2016), as described elsewhere.^31,33^ For each ROI, we averaged blood oxygen level-dependent (BOLD) signal fluctuations across voxels within the ROI and correlated this aggregate time series with BOLD signals of all brain voxels. This process generated 1000 Pearson correlation coefficients per voxel, i.e. one for each GSP1000 subject, which were then Fischer *z*-transformed and averaged across subjects to create a functional connectivity map per ROI. Functional connectivity map computations were performed using the ROI masks as seeds in the ‘connectome mapper’ function of Lead-DBS (lead-dbs.org).^34^ Subsequently, ROI-level fLNM scores were calculated by averaging positive Pearson correlation coefficients within the WMH mask, reflecting each ROI’s functional connectivity to WMH.

Structural lesion network mapping (sLNM) was performed using a normative structural connectome of 32 subjects of the Human Connectome Project (HCP32).^27^ The structural connectome was reconstructed by applying DSI Studio on multi-shell diffusion MRI data acquired on a MRI scanner specifically designed for high-fidelity connectome reconstruction. Streamlines resulting from whole brain tractography were normalized to MNI and aggregated across subjects.^35^ Using Lead-DBS, voxel-wise structural connectivity maps were computed per atlas ROI, quantifying per voxel the number of streamlines connecting the voxel to the ROI.^34^ ROI-level sLNM scores, reflecting structural connectivity between WMH and individual ROI, were determined by averaging the voxel values (representing streamline counts to the ROI) within the WMH mask.

Summarized, LNM yielded both a fLNM and sLNM score for each ROI per subject, indicating the functional and structural connectivity between WMH and ROI, respectively. In accordance with previous studies, we interpret these measures of WMH connectivity as indirect measures of disconnectivity.^11,13^

### Predictive modelling analysis

To evaluate the predictive capacity of fLNM and sLNM scores, we performed a predictive modelling analysis leveraging scikit-learn (v. 1.0.2, scikit-learn.org) and julearn (v. 0.3.0, juaml.github.io/julearn).^36,37^ This work defines ‘prediction’ in accordance with previous studies as the estimation of target variables using a trained statistical model on new unseen data—emphasizing the crucial aspect of model generalizability.^11,38,39^ We note that this definition varies from those indicating longitudinal study designs used in epidemiological contexts.^40^ In the analysis, six different feature sets were compared: (i) demographics (age, sex and education); (ii) total WMH volume + demographics; (iii) tract-level WMH volumes + demographics; (iv) ROI-level fLNM scores + demographics; (v) ROI-level sLNM scores + demographics; and (vi) ROI-level fLNM and sLNM scores + demographics.

For each cognitive domain, multivariable ridge regression models were trained using the abovementioned feature sets to predict cognitive domain scores. Ridge regression models include a L2 regularization, which is a technique to control the model complexity. It reduces coefficients to mitigate overfitting and to address multicollinearity improving the model’s ability to generalize to unseen data. We optimized the L2 regularization through a 10-fold nested cross-validation, tuning α-values ranging from 0.001 to 1000 (α = 0.001, 0.01, 0.1, 1, 10, 100, 1000). In this procedure, the α-values were optimized within an inner cross-validation loop, while the performance of the optimized models was evaluated in an outer loop based on test data not seen during training. This method prevents bias in the assessment of predictive performance. The model performance was scored by the Pearson correlation between actual and predicted cognitive domain scores, supplemented with explained variance (R^2^, coefficient of determination) and negative mean squared error as additional measures of performance. In line with best practices, explained variance was calculated via sum-of-squares formulation (using scikit-learn’s *r2_score*) instead of squaring Pearson correlations.^38^ Before model fitting, continuous input features were *z*-scored in a cross-validation consistent manner to avoid data leakage.^41^ To maintain a consistent distribution of the target variable across training and test sets, we employed julearn’s ‘ContinuousStratifiedKFold’ function for creating the folds. Cross-validations were repeated 10 times with varied random splits to minimize bias from any single split.^42^ This approach yielded 100 scores for each feature-target set combination, which were compared between feature sets using a machine learning-adjusted *t*-test.^43^ We repeated the predictive modelling analysis for different sample sizes (20%–100%, 1% steps, randomly sampled) to examine the robustness and sample size dependency of predictive performances. As a whole, this analysis follows current best practices of predictive modelling in neuroimaging to address overfitting as well as prevent leakage and circularity, ensuring accurate estimates of predictive validity.^38^

### Region of interest-level inferential statistics

To investigate whether WMH connectivity of specific brain circuits link to impaired cognitive performance, we conducted permutation-based testing for linear associations between regional LNM scores and cognitive domain scores in a general linear model. All statistical analyses were conducted in FSL’s Permutation Analysis of Linear Models (PALM) based on MATLAB (v. 2021b) and Python 3.9.1 leveraging neuromaps (v. 0.0.5).^44-46^ Statistical tests were two-sided (*n*_permutation_ = 5000), with a *P* < 0.05 as the significance threshold. To account for multiple comparisons, *P-*values were adjusted for family-wise error rate. General linear models were adjusted for age, sex and education. To obtain standardized β-coefficients, input variables were *z*-scored beforehand. As a result, β-coefficients and *P*-values were obtained for each cortical, subcortical and white matter ROI (*n*_ROIs_ = 480) indicating the strength and significance of the LNM score’s linear association with cognitive domain scores for each ROI. To aid in interpreting the spatial effect patterns, we averaged the β-coefficients representing cortical effects in the seven intrinsic resting state networks (Yeo networks), which reflect the cerebral cortex’s intrinsic functional organization.^33^ The Schaefer400 × 7 Atlas assigns ROIs to these networks: visual, somatomotor, dorsal attention, ventral attention (salience), limbic, frontoparietal control and default mode network.^29^ Significance was tested via spin permutations (*n*_spins_ = 1000), which represent a null model preserving the inherent spatial autocorrelation of cortical information.^47^

### Sensitivity analyses

To examine if our results are driven by specific analysis design decisions, we performed multiple sensitivity analyses.

During computations of fLNM scores, we decided to only consider positive Pearson correlations of resting state BOLD signal within WMH masks following previous approaches, as the role of negative correlations is controversial.^48^ However, some studies suggest biological meaning in anticorrelations of BOLD signal fluctuations.^49,50^ Hence, we conducted a sensitivity analysis based on fLNM scores computed by averaging only the negative Pearson correlations in the WMH masks. We reconducted the predictive modelling analysis and ROI-level inferential statistics using these negative fLNM scores.

Moreover, previous work employs thresholding to discard potentially noisy connectivity information. To further examine the effect of thresholding on our results, we repeated the predictive modelling analysis comparing the main analysis results to fLNM and sLNM scores computed based on 25% and 50% highest voxel intensities in the WMH mask. For negative fLNM scores, the lowest 25% and 50% voxel intensities in the WMH mask were considered.

Furthermore, we tested the robustness of predictive performance across different Schaefer Atlas resolutions. Therefore, we computed LNM scores for the Schaefer100 × 7 and Schaefer200 × 7 Atlas and repeated the predictive modelling analysis based on these measures.

Last, we tested *post hoc* whether the results regarding language performance prediction could be confounded by disconnection originating from the right hemisphere. Hence, we examined if language prediction remained stable considering only left-hemispheric WMH for the LNM score computations and left-hemispheric ROIs in the predictive modelling analysis.

### Supplementary analyses

Further analyses including LNM informed by the WMH penumbra,^51,52^ investigation of structure-function coupling of LNM scores, voxel-level lesion network maps and voxel-based lesion-symptom mapping^53,54^ are described in the Supplementary material, ‘Supplementary text S2’ section.

## Results

### Sample characteristics

The pooled study sample of 3485 patients had a mean age of 71.7 ± 8.9 years and 49.8% were female. Among patients, 777 (22.3%) had subjective cognitive impairment, 1389 (39.9%) had mild cognitive impairment, and 1319 (37.9%) had dementia. Further details on the sample characteristics can be found in Table 1. A heat map of WMH distribution can be found in Supplementary Fig. 3.

**Table 1:** Sample characteristics

Demographics	
Age in years, mean ± SD (*n*)	71.71 ± 8.87 (3485)
Female, *n* (%)	1737 (49.8)
Years of education, mean ± SD (*n*)	12.89 ± 4.45 (3485)
**Patient ethnicity**	
Afro-Caribbean, *n* (%)	198 (5.7)
Asian, *n* (%)	237 (6.8)
Caucasian/European/White, *n* (%)	1620 (46.5)
Hispanic, *n* (%)	146 (4.2)
Other, *n* (%)	52 (1.5)
**Diagnosis**	
Subjective cognitive impairment, *n* (%)	777 (22.30)
Mild cognitive impairment, *n* (%)	1389 (39.86)
Dementia, *n* (%)	1319 (37.85)
**For dementia cases: probable aetiology**	
Alzheimer’s dementia, *n* (%)	764 (57.9)
Vascular dementia, *n* (%)	85 (6.4)
Frontotemporal dementia, *n* (%)	44 (3.3)
Dementia with Lewy bodies, *n* (%)	24 (1.8)
**Cardiovascular risk factors**	
Current smoking, *n* (%)	499 (14.3)
Previous smoking, *n* (%)	459 (13.2)
Hypertension, *n* (%)	1714 (49.2)
Hypercholesterolemia, *n* (%)	1098 (31.5)
Diabetes mellitus, *n* (%)	492 (14.1)
BMI, mean ± SD (*n*)	25.28 ± 4.75 (640)
**Comorbidities**	
Atrial fibrillation, *n* (%)	98 (2.8)
History of prior stroke, *n* (%)	244 (7.0)
History of prior transient ischaemic attack (TIA), *n* (%)	62 (1.8)
History of prior other vascular events, *n* (%)	715 (20.5)
**Imaging**	
WMH volume in ml, median [IQR] (*n*)	6.19 [14.21] (3485)
**Cognitive function**	
Mini-Mental State Examination, mean ± SD (*n*)	25.0 ± 4.7 (3327)
Attention/executive function, *z*, mean ± SD (*n*)	−1.12 ± 1.10 (3446)
Information processing speed, *z*, mean ± SD (*n*)	−0.96 ± 1.61 (2417)
Language, *z*, mean ± SD (*n*)	−1.08 ± 1.86 (2041)
Verbal memory, *z*, mean ± SD (*n*)	−1.48 ± 1.30 (3242)

BMI = body mass index; IQR = interquartile range; SD = standard deviation; WMH = white matter hyperintensities of presumed vascular origin; *z* = harmonized *z*-score.

### Predictive modelling analysis

**Figure 2:**
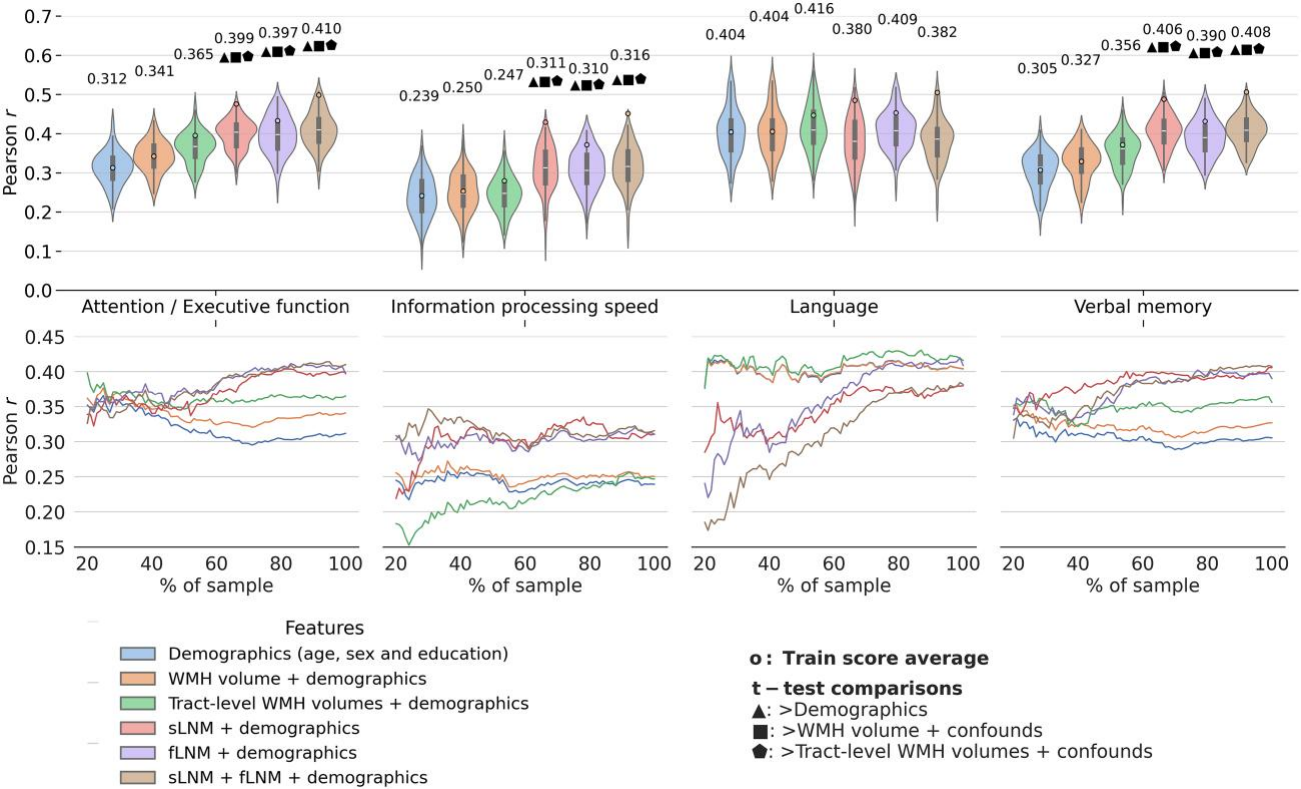
**Predictive modelling analysis.** Violin plots illustrate prediction outcomes across cognitive domains. Each violin displays the distribution of Pearson correlations (between actual and predicted cognitive domain performance; 10-fold cross-validation × 10 repeats = 100 folds → 100 Pearson correlations) for a model informed by a different feature set. The higher the Pearson correlation, the higher the prediction performance. Blue = demographics (age, sex and education); orange = total WMH volume + demographics; green = tract-level WMH volumes + demographics; red = sLNM scores + demographics; purple = fLNM scores + demographics; brown = sLNM scores + fLNM scores + demographics. Average Pearson correlations are indicated above each violin, with coloured dots showing training score averages. Geometric symbols denote *t*-test results comparing LNM-based models against demographics and WMH volume-based models: filled triangle ▴ indicates significantly higher Pearson correlation than demographics, filled square ▪ indicates significantly higher Pearson correlation than WMH volume + demographics; and filled pentagon ⬟ indicates significantly higher Pearson correlation than tract-level WMH volume + demographics. Below the violin plots, performance curves display the average Pearson correlations across folds, for subsets randomly sampled in sizes ranging from 20% to 100% of the entire dataset. Line colours match the corresponding violin plots in **A**, which display predictive modelling results for the full sample size. Again, higher Pearson correlation indicates higher prediction performance. fLNM = functional lesion network mapping; sLNM = structural lesion network mapping; WMH = white matter hyperintensities of presumed vascular origin.

To evaluate if information on WMH network connectivity exceeds the predictive capacity of volumetric WMH metrics for cognitive performance, we first computed regional fLNM and sLNM scores, that capture the functional and structural connectivity profile of WMH. We then employed ridge regression for predictive modelling. Model performance was assessed via Pearson correlation (*r*) of predicted and actual cognitive domain scores averaged across folds. All models incorporated age, sex and education (demographics) as features to establish a performance baseline. The corresponding results are visualized in Fig. 2A. In summary, compared to WMH volumes, LNM scores significantly improved cognitive function prediction in all domains, except language. In detail, the predictive performance achieved by the demographics-only model was *r* = 0.312 for attention/executive function, *r* = 0.239 for information processing speed, *r* = 0.404 for language and *r* = 0.305 for verbal memory. Models informed by total or tract-wise WMH volumes achieved a predictive performance of *r* = 0.341–0.365 for attention/executive function, *r* = 0.247–0.250 for information processing speed, *r* = 0.404–0.416 for language and *r* = 0.327–0.356 for verbal memory. For the prediction of attention/executive function, models informed by LNM scores exhibited a significantly higher predictive performance than models informed by volumetric WMH measures (LNM: *r* = 0.399–0.410 versus WMH volume: *r* = 0.341–0.365; adjusted *t*-test, all *P* < 0.05). LNM-informed models also better predicted information processing speed (LNM: *r* = 0.310–0.316 versus WMH volume: *r* = 0.247–0.250, adjusted *t*-test, all *P* < 0.05) as well as verbal memory (LNM: *r* = 0.390–0.408 versus WMH volume: *r* = 0.327–0.356; adjusted *t*-test, all *P* < 0.05). Across these domains, the best prediction was achieved by models incorporating both structural and functional LNM scores. For attention/executive function, comparing the improvement from the demographics-based model to the model informed by total WMH volume (0.341 − 0.312 = 0.029) with the improvement to the model based on both LNM modalities (0.410 − 0.312 = 0.098), the usage of fLNM and sLNM scores amounts to a 3.38-fold increase (0.098/0.029 = 3.38) in added predictive performance. Considering both LNM modalities for predicting information processing speed and verbal memory amounted to 7.00-fold and 4.68-fold increase in predictive performance, respectively. For the prediction of language domain scores, performance between LNM-informed models and WMH volume measures did not differ significantly (LNM: *r* = 0.380–0.409 versus WMH volume: *r* = 0.404–0.416, all *P* > 0.05). See Supplementary material, Sections S4 and S5 for predictive modelling results using explained variance and negative mean squared error as scoring methods. Details on regional averages of LNM scores are shown in Supplementary Fig. 6.

To test the robustness of prediction results, we repeated the analysis in randomly chosen subsamples of increasing sizes (Fig. 2B). For attention/executive function and verbal memory, LNM-informed models started to consistently exceed WMH volume-based models at ∼50% (attention/executive function: *n* = 1723, verbal memory: *n* = 1712; note that data availability differed between cognitive domain scores) of the sample size. For information processing speed, LNM-informed models surpassed WMH volume-based models at ∼25% (*n* = 604) of the sample size. Regarding language, LNM-informed models approximated the performance of WMH volume-based models with increasing sample sizes. For all cognitive domain scores, predictive performance in the sample size range 80–100% showed high stability and only minor increases indicating saturation.

### Contextualization of WMH connectivity: region of interest analysis

We tested if WMH connectivity to specific brain circuits links to cognitive performance by quantifying the association between regional LNM scores (grey matter regions and white matter tracts) and cognitive domain scores adjusting for age, sex and education.

**Figure 3:**
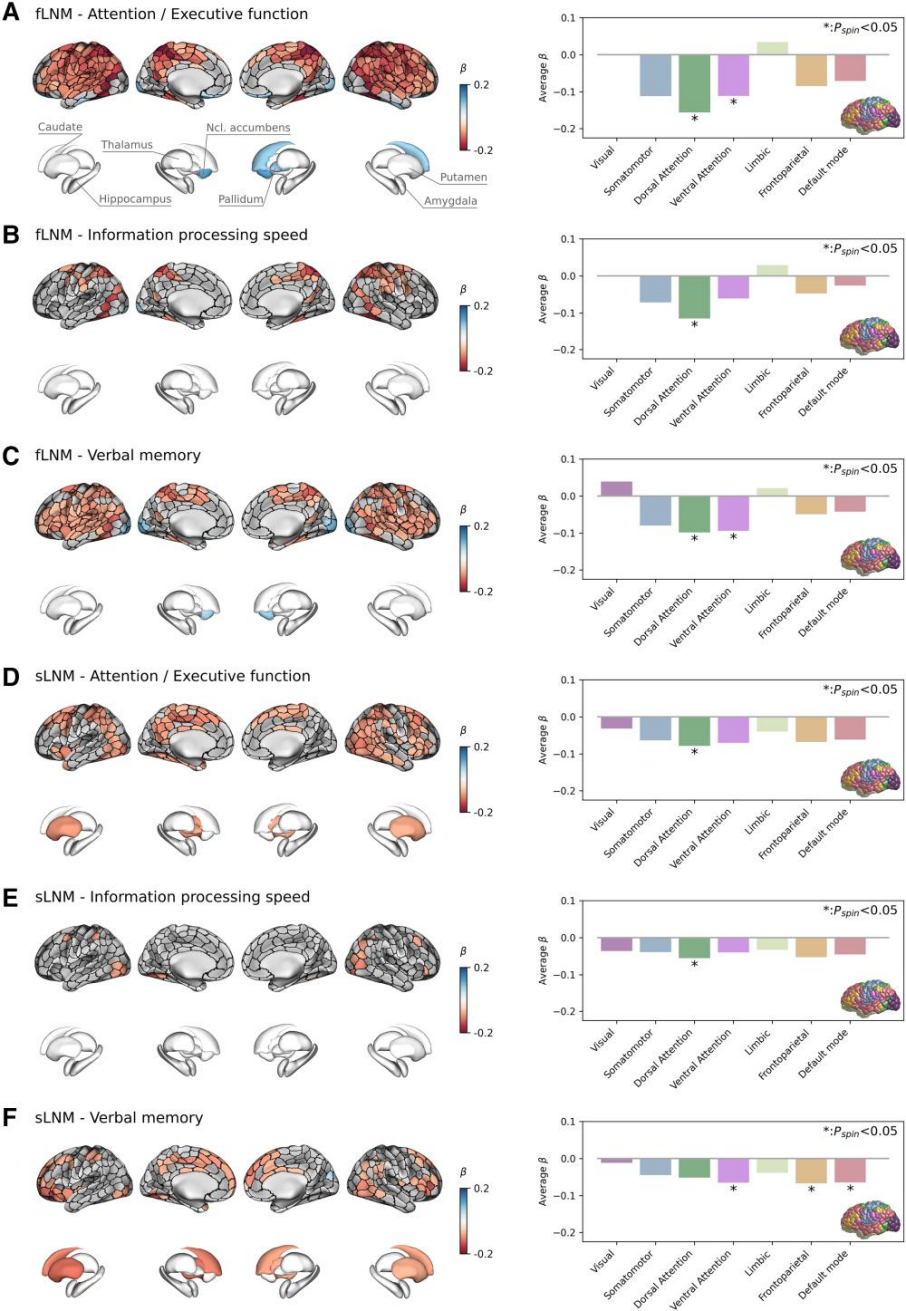
**Inferential statistics results of cortical and subcortical grey matter.** Anatomical plots on the *left* display the regional relationship between lesion network mapping (LNM) scores and cognitive domain scores. Regions of interest (ROIs) in which LNM scores across participants were significantly associated with cognitive domain scores after family-wise error rate correction are highlighted by colours encoding β-coefficients from general linear models: a negative β (red) denotes that a higher regional LNM score, i.e. higher WMH connectivity, is associated to a lower performance in individual cognitive domains; a positive β (blue) indicates that a higher regional LNM score is linked to a higher cognitive domain performance. Bar plots on the *right* display the corresponding β*-*coefficients averaged in the canonical (Yeo) resting state functional connectivity networks. The brain in the *bottom right* indicates the regional distribution of the canonical resting state networks with colours corresponding to the bars. Statistical significance was assessed using spin permutations. Each row corresponds with a different combination of lesion network mapping modality and cognitive domain: (**A**) fLNM—attention/executive function; (**B**) fLNM—information processing speed; (**C**) fLNM—verbal memory; (**D**) sLNM—attention/executive function; (**E**) sLNM—information processing speed; and (**F**) sLNM—verbal memory. fLNM = functional lesion network mapping; *P_spin_* = *P*-value derived from spin permutations; sLNM = structural lesion network mapping; WMH = white matter hyperintensities of presumed vascular origin.

Results of the general linear model linking LNM scores in cortical and subcortical grey matter regions to cognitive domain scores are shown in Fig. 3. Higher fLNM scores (i.e. increased WMH connectivity) in cortical regions of the dorsal attention and ventral attention networks were linked to lower attention/executive function and verbal memory (Fig. 3A and C). Regarding information processing speed, the extent of the effect was limited to several cortical brain areas mapping to the dorsal attention network (Fig. 3B). In terms of sLNM, higher scores in the dorsal attention network were significantly associated with lower attention/executive function and information processing speed (Fig. 3D and E). Again, information processing speed showed a spatially more limited effect pattern. The relationship of regional sLNM and verbal memory scores showed a different spatial distribution mapping to the ventral attention, frontoparietal and default mode network (Fig. 3F). The cortical and subcortical LNM scores showed no significant association with the language domain score.

**Figure 4:**
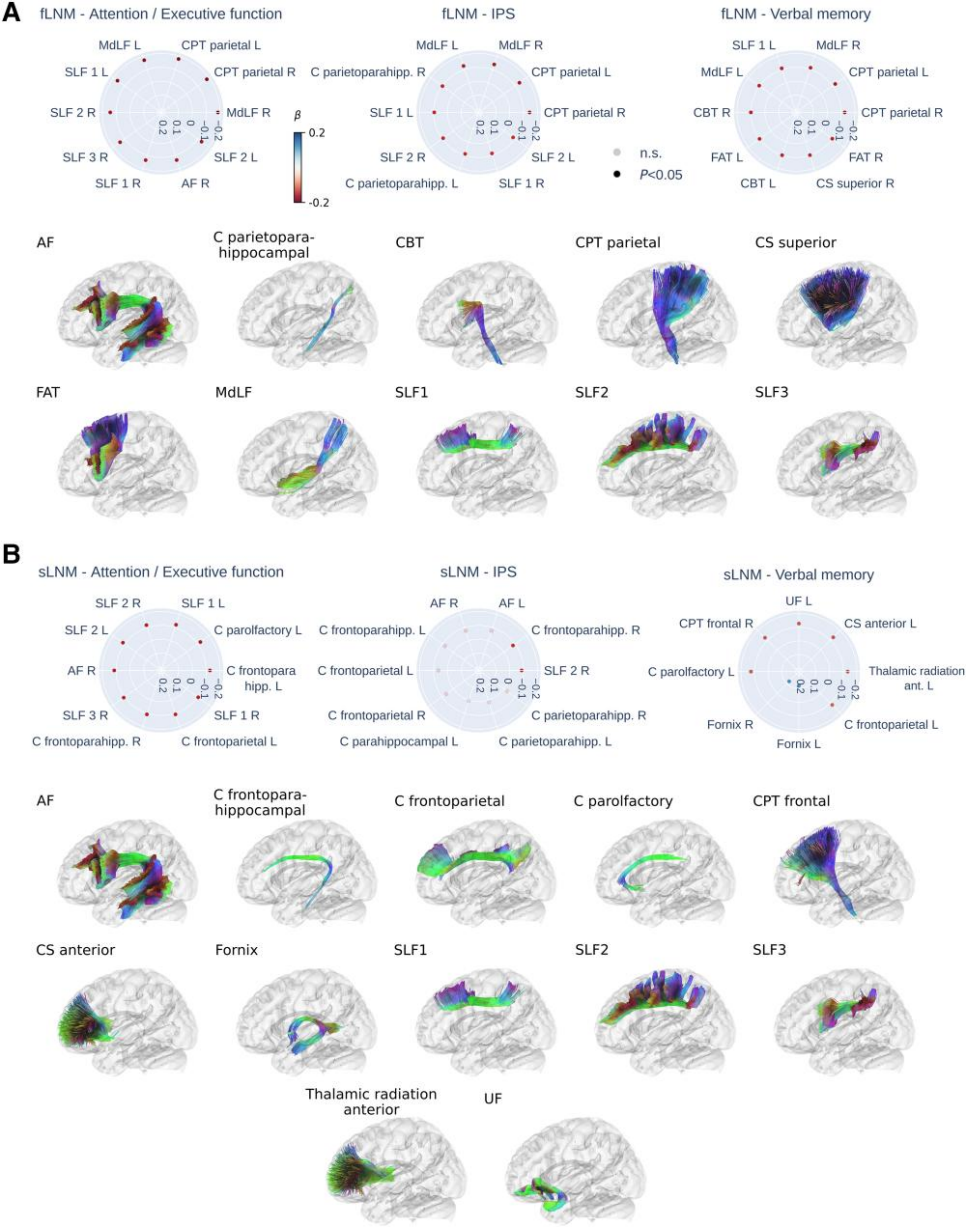
**Inferential statistics results of white matter tracts.** Radar plots displaying the top 10 strongest linear associations (standardized β) for the functional (**A**) and structural (**B**) lesion network mapping (LNM) scores in each tract in association with cognitive domain scores. Strongest associations are shown at the 3 o’clock position, decreasing in strength counterclockwise. Red dots indicate a negative association (higher LNM score − lower cognitive domain score) and blue dots indicate a positive association (higher LNM score − higher cognitive domain score). Faintly coloured dots indicate non-significant associations. Tracts with a significant association are displayed below the radar plots in alphabetical order. For paired tracts only left side examples are visualized. AF = arcuate fascicle; C = cingulate; CBT = corticobulbar tract; CPT = corticopontine tract; CS = corticostriatal pathway; F = fornix; FAT = frontal aslant tract; MdLF = middle longitudinal fasciculus; SLF = superior longitudinal fasciculus; UF = uncinate fasciculus; fLNM = functional lesion network mapping; IPS = information processing speed; n.s. = non-significant; *P* = *P*-value; sLNM = structural lesion network mapping.

The results for anatomically predefined white matter tracts are shown in Fig. 4. For tract-level fLNM, lower cognitive performance in attention/executive function, information processing speed and verbal memory was most strongly linked to higher fLNM scores in association and projection tracts connecting the parietal cortex (Fig. 4B): the middle longitudinal fasciculus (MdLF), parietal corticopontine tract (CPT), dorsal, medial and ventral sections of the superior longitudinal fasciculus (SLF 1–3), the parietoparahippocampal cingulate (C parietoparahipp.). For attention/executive function, a significant negative effect was also evident for the right arcuate fasciculus (AF). For verbal memory, significant negative effects were additionally found for the corticobulbar tract (CBT) and frontal aslant tract (FAT).

Regarding tract-level sLNM, lower attention/executive function and verbal memory were significantly associated with higher sLNM scores in association and projection tracts connecting frontal regions (Fig. 4C): the frontoparahippocampal cingulate (C parietoparahipp.), parolfactory cingulate (C parolfactory), the superior longitudinal fasciculus (SLF 1–3), frontoparietal cingulate (C frontoparietal), anterior thalamic radiation, anterior corticostriatal pathways (CS anterior), uncinate fascicle, frontal corticopontine tract (CPT frontal). For attention/executive function, a significant negative effect was also evident for the right AF. Furthermore, higher verbal memory scores were significantly linked to higher sLNM scores in the fornices. Information processing speed showed a significant negative association with sLNM scores in the right medial superior longitudinal fasciculus (SLF 2) and frontoparahippocampal cingulate (C frontoparahipp.). Tract-level LNM scores showed no significant association with language function. For plots displaying all tract-level associations refer to Supplementary Figs 7 and 8.

The spatial effect patterns, i.e. β-coefficient maps, showed considerable overlap with 26 of 28 effect pattern pairs being significantly correlated (see Supplementary Fig. 9 for a correlation matrix).

### Sensitivity analyses

Predictive modelling results were stable when using negative fLNM scores (based on anti-correlations in resting state fMRI measures) and when including a 25% or 50% thresholding step (Supplementary Fig. 10). Exploratory ROI-level inferential statistics based on negative fLNM scores indicated that lower attention/executive function and information processing speed were more significantly associated with more negative fLNM scores in the default mode network (Supplementary Figs 11 and 12). Predictive modelling results were also robust if considering the Schaefer100 × 7 and Schaefer200 × 7 Atlas during LNM score computations instead of the Schaefer400 × 7 Atlas (Supplementary Fig. 13). Of note, models informed by Schaefer100 × 7 and Schaefer200 × 7 fLNM scores and demographics predicted language function significantly better than models informed by demographics and WMH volume measures. Predictive modelling of language performance only informed by left-hemispheric LNM scores showed stable results (Supplementary Fig. 14). In sum, our results were robust across sensitivity analyses.

### Supplementary analyses

Supplementary analyses are detailed in the Supplementary material, ‘Supplementary text S2’ section. WMH penumbra-based LNM scores slightly improved predictive performance compared to original LNM scores for attention/executive function (*r* = 0.406–0.416), information processing speed (*r* = 0.318–0.328) and verbal memory (*r* = 0.402–0.414) (Supplementary Fig. 15). Functional and structural LNM scores were significantly correlated across ROIs and across subjects (Supplementary Fig. 16) with a mean correlation coefficient of 0.50 ± 0.26. Voxel-level lesion network maps indicating white matter regions that contribute to variance in cognitive domain function are shown in Supplementary Figs 17 and 18. Voxel-based lesion-symptom mapping based on sparse canonical correlation analysis revealed significant voxel-level associations between WMH occurrence and cognitive performance across domains. Significant associations were located bilaterally in periventricular regions for all cognitive domains. The corresponding lesion-symptom maps can be found in Supplementary Fig. 19. Average prediction performance achieved by voxel-based lesion-symptom mapping was lower compared to LNM-informed models: average *r* = 0.250 for attention/executive function, *r* = 0.196 for information processing speed, *r* = 0.204 for language and *r* = 0.310 for verbal memory (Supplementary Fig. 20).

## Discussion

In a large multicentric sample of memory clinic patients, we conducted an in-depth examination of the link between functional and structural LNM scores and cognitive performance. We report two main findings: (i) both structural and functional LNM scores, capturing WMH connectivity, significantly improved the prediction of cognitive performance compared to WMH volume in the domains attention/executive function, information processing speed and verbal memory; and (ii) WMH connectivity associated with lower cognitive performance, predominantly mapped to the dorsal and ventral attention networks.

### LNM scores surpass WMH volumes in predicting cognitive performance

In current clinical practice, cognitive impairment is often attributed to cerebrovascular disease on the basis of total WMH burden assessed through visual inspection, but interindividual variance in this relationship can lead to diagnostic dilemmas. Previous lesion-symptom mapping studies have demonstrated that strategic WMH locations, specifically in commissural and association tracts, are statistically more likely to be associated with lower cognitive performance.^4,6,7^ Our LNM-approach adds to this perspective, not only considering the location of WMH but also integrating them with network connectivity information to capture the WMH network embedding. In our analysis, statistical models capitalizing on LNM scores demonstrated superior performance over those relying on total or tract-level WMH volume in predicting cognitive performance in almost all cognitive domains. Therefore, LNM could be leveraged for improving out-of-sample prediction of cognitive performance over demographics, total WMH volume and strategic WMH location in specific white matter tracts.

Of note, the prediction of cognitive performance improved marginally by adding WMH volumes alone.^38^ Comparing the improvement of imaging-informed models, the usage of fLNM and sLNM scores yielded to a 3- to 7-fold increase in added predictive performance over demographics across the three cognitive domains. Our findings are important, given the longstanding reliance on WMH extent as a primary imaging surrogate marker for cognitive impairment in CSVD. We provide evidence for the role of WMH-related ‘covert’ network-level effects of chronic vascular injury in cognitive deficits, as indicated previously in studies from smaller clinical or population-based studies.^9,55-57^ However, the overall amount of variance explained by WMH, even with the use of network-level metrics, was small (all R^2^ < 0.2, Supplementary Fig. 4) and unexplained variance in cognitive performance remains, likely due to factors beyond the scope of our study, such as genetic predispositions for neurodegeneration, individual brain network resilience to chronic injury, accumulation of misfolded proteins and changes in brain morphology like cortical atrophy.

Improved prediction of cognitive performance was achieved irrespective of the applied LNM modality. Contrasting prior studies suggesting the inferiority of functional LNM compared to structural approaches for predicting cognitive performance post-stroke,^11,58^ our contrary findings might arise from differences in the LNM approach as well as our focus on WMH rather than ischaemic stroke lesions. The ROI-based functional LNM method we used may be more suitable to detect the widespread network disturbances induced by WMH, as opposed to the localized impact of stroke lesions. Notably, fLNM and sLNM scores were positively correlated, suggesting some degree of structure-function coupling that could account for their comparable predictive performance. However, the correlation strength was mostly moderate and prediction performance of fLNM and sLNM differed noticeably across sample sizes. In addition, among LNM-informed models, those incorporating both fLNM and sLNM modalities yielded the strongest results. This suggests that both LNM approaches are equally valuable for achieving a high predictive accuracy in general but might also offer complementary information.

Remarkably, including LNM scores based on both WMH and adjacent normal-appearing white matter—the so-called WMH penumbra—improved predictive performance (Supplementary Fig. 15). This suggests that white matter abnormalities beyond visible lesions contribute to cognitive variance in memory clinic patients, reflecting the notion that cerebrovascular pathology contributing to cognitive impairment is widespread and diffuse.^1^ Future analyses leveraging CSVD imaging features beyond WMH should expand on this finding.

Although prediction of almost all cognitive domains was improved by LNM scores, predictive performance for language functions did not exceed that of WMH volumes and demographics. From a network perspective, we argue that this finding might be explained by the relatively confined network of left-lateralized brain regions involved in language functions, which might present lower vulnerability to WMH disconnectivity compared to cognitive functions, such as information processing speed, that rely on a widely distributed network of brain regions.^59^ Hence, analyses including patient-level information on structural and functional connectivity that can account for interindividual variability in network configuration should expand on this result. Notably, our sensitivity analyses showed that fLNM scores based on other Schaefer Atlas resolutions (100 × 7, 200 × 7) significantly improved language prediction compared to models based on demographics and total WMH volume. This may be because these resolutions better identify language-relevant areas, or more technically, due to lower dimensionality with fewer ROIs, which reduces overfitting. In sum, the slight improvement of WMH-based measures over demographic-based predictions suggests that WMH contribute to a limited extent to variance in language function.

### WMH related to cognitive impairment map to attention control networks

WMH are considered to compromise cognitive performance by impacting the function of specific brain networks.^10^ To localize these effects, we investigated regional associations between functional and structural LNM scores to cognitive performance. We found that higher LNM scores of cortical areas of the dorsal and ventral attention networks were linked to lower attention and executive function, information processing speed and verbal memory (Fig. 3). Therefore, higher WMH connectivity in these networks is associated with reduced cognitive performance indicating that WMH impair cognitive function by disrupting the respective connecting white matter fibre tracts.

The dorsal attention network—including the frontal eye field, the superior parietal lobule, the intraparietal sulcus and caudal areas of the medial temporal gyrus—governs top-down attention control by enabling voluntary orientation, with increased activity in response to cues indicating the focus location, timing or subject.^60,61^ The ventral attention network comprises the ventrolateral frontal cortex, medial areas of the superior frontal cortex and the temporoparietal junction.^49,62^ This system exhibits activity increases during bottom-up attention control, i.e. upon detection and orientation to salient targets, especially when they appear in unexpected locations.^60,63^ As the effect patterns largely converged on these networks (Supplementary Fig. 9), we argue that WMH affect the cognitive functions emerging from these networks, specifically top-down and bottom-up attention control. This aligns with the observation that deficits in attention and executive function are among the most prominent symptoms in CSVD and VCI in general.^1^ Furthermore, prior work demonstrates altered resting state functional connectivity as well as task activation in attention control networks related to CSVD.^10,64,65^ Given the covariance of the identified effect patterns, we speculate that WMH contribute to variance in the performance of other cognitive domains, e.g. information processing speed, by affecting the attention demands posited by the corresponding tests.

### WMH contribute to cognitive impairment by disrupting frontal and parietal white matter tracts

Regional findings in grey matter areas of the attention control networks are further complemented by white matter tract-level results (Fig. 4). Functional and structural LNM converged on a significant involvement of tracts connecting frontal and parietal areas involved in attention: the dorsal, medial and ventral section of the SLF—which are known to connect the anterior and posterior parts of the dorsal and ventral attention networks, the medial longitudinal fasciculus, the corticopontine tract, frontoparietal sections of the cingulate, the anterior thalamic radiation, the frontal aslant tract and the arcuate fascicle. Although there were some differences in highlighted tracts between functional and structural LNM, this possibly reflects that both approaches capture different aspects of the same anatomy, with sLNM possibly being more sensitive to direct WMH-induced disruption of axonal connections and functional LNM also reflecting effects mediated via polysynaptic brain circuitry.

Strikingly, in the context of verbal memory, structural WMH connectivity pinpointed a distinct set of memory-relevant tracts: the uncinate fascicle, cingulate and fornix. Intriguingly, disruptions in fornix connectivity due to WMH were associated with improved verbal memory in patients, a finding that appears counterintuitive given the fornix’s involvement in maintaining memory function. This paradox may be attributable to WMH disrupting inhibitory fibres.

### Lesion anticorrelations are associated with cognitive function

The attention control networks are functionally contrasted by the default mode network which shows, instead of being engaged during externally focused tasks, increased activity during internally directed attention and self-referential processes.^66^ As a result, the default mode network and the attention control networks are often found to be anticorrelated at rest.^49^ This anticorrelation is thought to reflect a fundamental aspect of brain organization and the complex dynamic interplay between the networks is thought to be central for cognitive processing. Resting state fMRI studies in CSVD patients suggest that WMH might affect the DMN and attention network interaction, particularly affecting anterior-posterior communication by disrupting long associative white matter fibre tracts.^10,64^ Our findings indicate that stronger anticorrelation between the default mode network and WMH—reflected by more negative fLNM scores—correlates with reduced attention, executive function and processing speed, supporting this hypothesis (Supplementary Figs 11 and 12). Furthermore, by demonstrating that prediction performance is stable if based on negative fLNM scores (Supplementary Fig. 10), our results underscore the notion of anticorrelations yielding biologically and clinically meaningful information.

### Clinical implications

Drawing upon a comprehensive LNM analysis in a memory clinic sample of patients with differing extent and aetiology of cognitive impairment, our research converges on a unifying hypothesis: WMH contribute to variance in cognitive functions by disrupting brain circuitry involved in attention control. Our findings not only shed light on the intricate relationships between CSVD, neuroanatomy and cognitive impairment, but they also hint at potential avenues of clinical utilization. The definitive role of CSVD treatments, particularly in precluding cognitive sequelae, is yet to be firmly established. Although there have been promising outcomes related to risk factor modification, particularly blood pressure control,^67,68^ pointing towards enhanced cognitive trajectories, clinical trials in VCI require biomarkers to robustly identify vascular contributions to cognitive impairment and vulnerable individuals. Integrating lesion connectivity information into clinical assessments could improve the diagnostic accuracy and help to distinguish between causes of cognitive impairment. Furthermore, leveraging connectivity information could facilitate the identification of subgroups at risk of cognitive disorders through vascular lesions likely to reap the most substantial benefits from medical interventions. As we progress, biomarkers targeting the brain networks affected by WMH could inform preventive and therapeutic interventions.

### Strengths and limitations

This study’s strength lies in its integration of innovative analytical techniques with a large, multicentric dataset.^69^ However, we acknowledge several limitations that warrant consideration when interpreting our findings. The inclusion of selected patient samples in several cohorts may limit generalizability to the broader memory clinic population. Additionally, with most patients being of European ancestry, the generalizability of our findings to other ethnicities remains to be established. Furthermore, despite the harmonization of cognitive and imaging data, biases stemming from variations in data acquisition and processing protocols across sites may have impacted our results. On a technical note, while computing fLNM scores, we sampled resting state BOLD signals in the white matter, typically regarded as noisy and often dismissed as an artefact. However, by integrating it with WMH data, we successfully predicted cognitive performance and demonstrated correlations with structural connectivity information. This challenges the traditional view of the white matter BOLD signal as a mere artefact and supports recent studies—including LNM analyses of white matter lesions in multiple sclerosis—demonstrating that it contains biologically meaningful information.^70-72^

## Conclusion

WMH-related brain network connectivity measures significantly improve the prediction of current cognitive performance in memory clinic patients compared to WMH volume or epidemiological factors. Our findings highlight the contribution of WMH disconnectivity, particularly in attention-related brain regions, to vascular cognitive impairment. As this research field progresses, harnessing neuroimaging markers of WMH connectivity in CSVD has the potential to aid individualized diagnostic and therapeutic strategies.

## Supplementary Material

awae315_Supplementary_Data

## Data Availability

Analysis code can be accessed on GitHub (https://github.com/csi-hamburg/2024_petersen_wmh_disconnectivity_memory_clinic). The data that support the findings of this study are available from the project leads on reasonable request (https://metavcimap.org/group/become-a-member/). Restrictions related to privacy and personal data sharing regulations and informed consent may apply.
