# Supplementary material for: Enhancing cognitive performance prediction by white matter hyperintensity connectivity assessment
Source: Brain. 2024 Oct 14;147(12):4265–79. doi: 10.1093/brain/awae315 (PMC11629703; doi:10.1093/brain/awae315)
Supplement: awae315_Supplementary_Data [file awae315_supplementary_data.pdf]

Enhancing Cognitive Performance Prediction by White Matter Hyperintensity Connectivity Assessment: A Multicenter Lesion Network Mapping Analysis of 3,485 Memory Clinic Patients

Supporting Information

Marvin Petersen, Mirthe Coenen, Charles DeCarli, Alberto de Luca, Ewoud van der Lelij, Alzheimer's Disease Neuroimaging Initiative, Frederik Barkhof, Thomas Benke, Christopher P. L. H. Chen, Peter Dal-Bianco, Anna Dewenter, Marco Duering, Christian Enzinger, Michael Ewers, Lieza G. Exalto, Evan F. Fletcher, Nicolai Franzmeier, Saima Hilal, Edith Hofer, Huberdina L. Koek, Andrea B. Maier, Pauline M. Maillard, Cheryl R. McCreary, Janne M. Papma, Yolande A. L. Pijnenburg, Reinhold Schmidt, Eric E. Smith, Rebecca M. E. Steketee, Esther van den Berg, Wiesje M. van der Flier, Vikram Venkatraghavan, Narayanaswamy Venketasubramanian, Meike W. Vernooij, Frank J. Wolters, Xu Xin, Andreas Horn, Kaustubh R. Patil, Simon B. Eickhoff, Götz Thomalla, J. Matthijs Biesbroek, Geert Jan Biessels, Bastian Cheng

Content

Methods 3
Supplementary figure S1 – Investigated white matter tracts of the HCP1065 atlas 3
Supplementary text S2 - Supplementary analyses 4
Lesion network mapping informed by the WMH penumbra 4
Correlation of lesion network mapping scores 4
Voxel-level lesion network maps 4
Voxel-based lesion-symptom mapping 4
Results 6
Figure S3 – White matter hyperintensity distribution 6
Figure S4 – Predictive modeling analysis with explained variance (R^2, coefficient of determination) scoring 7
Table S5 – Predictive modeling analysis results – Average negative mean squared error 8
Figure S6 – Region of interest-level averages of lesion network mapping scores 9
Figure S7 – Tract-level functional lesion network mapping 10
Figure S8 – Tract-level structural lesion network mapping 11
Figure S9 – Spatial correlations of region of interest-level β coefficients 12
Figure S10 – Sensitivity analysis: Predictive modeling analysis 13
Figure S11 – Sensitivity analysis: Inferential statistics results of cortical and subcortical gray matter based on negative functional lesion network mapping scores 14
Figure S12 – Sensitivity analysis: Inferential statistics of white matter tracts based on negative functional lesion network mapping scores 16
Figure S13 – Sensitivity analysis: Predictive modeling on LNM scores based on different Schaefer Atlas resolutions 17
Figure S14 – Sensitivity analysis: Prediction of language function based on LNM scores of left-hemispheric WMH 18
Figure S15 – Predictive modeling based on WMH penumbra-informed LNM 19
Figure S16 – Structure-function correlations of regional lesion network mapping scores 20
Figure S17 - Voxel-level lesion network maps 21
Figure S18 - Voxel-level lesion network maps scaled by the white matter hyperintensity distribution map 22
Figure S19 - Voxel-level lesion-symptom maps 23
Figure S20 – Prediction performance of voxel-based lesion symptom mapping 25

Methods

Supplementary figure S1 – Investigated white matter tracts of the HCP1065 atlas

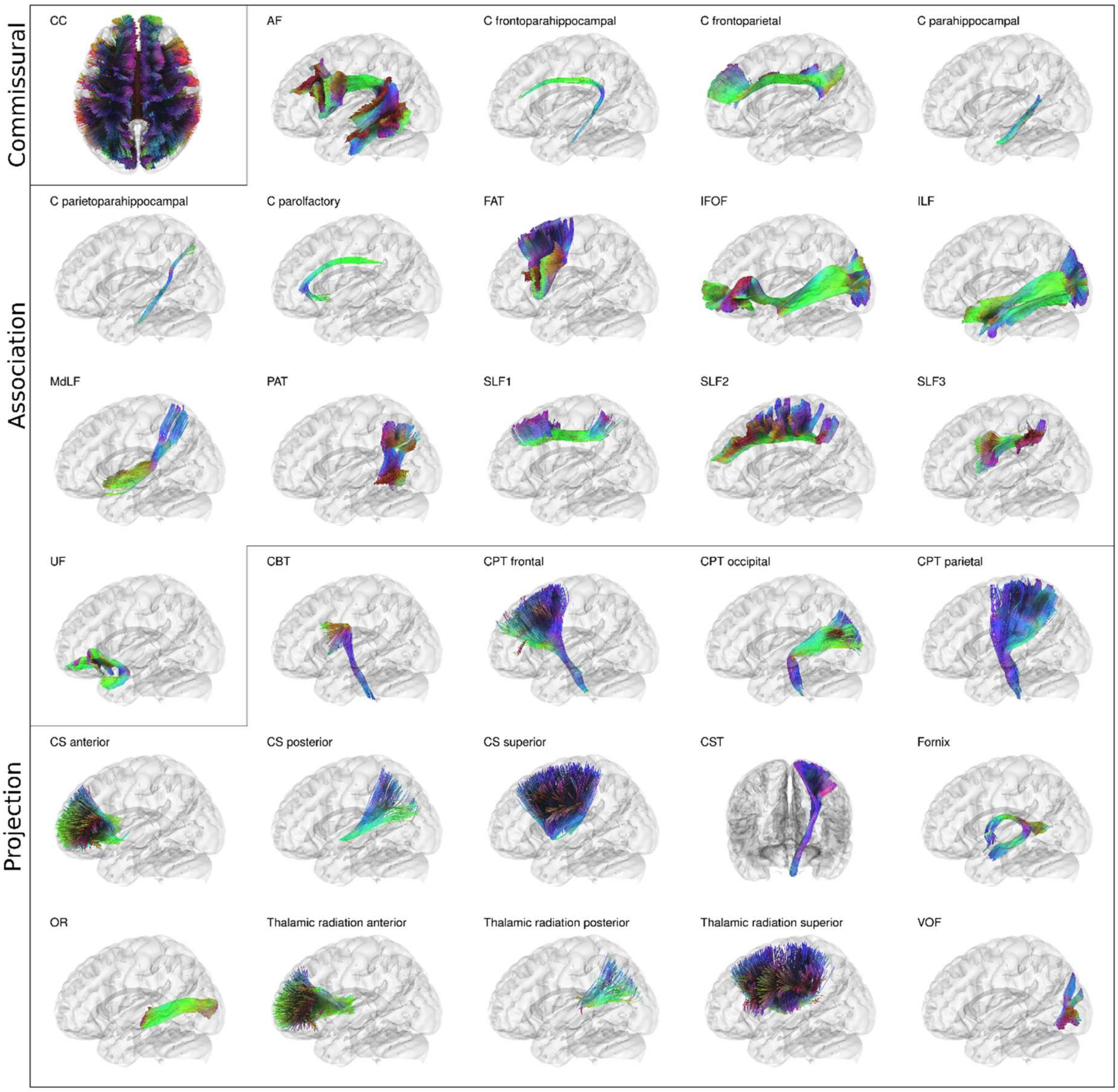

Anatomical depiction of the white matter tracts investigated, categorized into association, projection and commissural tracts. For paired tracts only left side examples are visualized. *Tract abbreviations*: Commissural tracts – CC = corpus callosum; Association tracts - AF = arcuate fascicle, C = cingulate, FAT = frontal aslant tract, IFOF = inferior fronto-occipital fasciculus, ILF = inferior longitudinal fasciculus, MdLF = middle longitudinal fasciculus, PAT = posterior aslant tract, SLF = superior longitudinal fasciculus, UF = uncinate fasciculus; Projection tracts – CBT = corticobulbar tract, CPT = corticopontine tract, CS = corticostriatal pathway, CST = corticospinal tract, FPT = frontopontine tract, F = fornix, OPT = occipitopontine tract, OR = optic radiation, VOF = Vertical occipital fasciculus.

Supplementary text S2 – Supplementary analyses

Lesion network mapping informed by the WMH penumbra.

Previous imaging analyses suggest that WMH may represent foci of more diffuse and widespread microstructural abnormalities of the white matter.[1,2] These findings have led to the hypothesis of a 'WMH penumbra' extending up to two millimeters into normal-appearing white matter.[3] To capture network effects associated with the WMH penumbra, we reperformed LNM score computations on WMH masks three-dimensionally dilated by 2mm. Subsequently, we repeated the predictive modeling analysis based on LNM scores from WMH and WMH penumbra.

Correlation of lesion network mapping scores

To test for a structure-function-coupling of lesion network mapping scores, we correlated functional and structural lesion network mapping scores 1) across subjects per region of interest and 2) across regions of interests per subjects. Corresponding results can be found in *supplementary figure S13.*

Voxel-level lesion network maps

We generated voxel-level lesion network maps to identify white matter areas crucial for cognitive performance. This involved averaging the voxel-level connectivity maps of the ROIs significantly linked to cognitive domain scores. These maps, created for each combination of the four cognitive domains and two LNM modalities, highlight regions where connectivity links to cognitive variance. We then scaled these maps by the WMH frequency map, which reflects the prevalence of WMH in each voxel across the analysis sample. The resulting maps reveal regions where WMH most commonly contribute to variance in cognitive performance. The maps can be found in *supplementary figures S14 & S15.*

Voxel-based lesion-symptom mapping

For our analysis, it is important to determine whether LNM provides predictive value for cognitive performance beyond lesion location alone. In the main analysis, we addressed this by incorporating models based on regional (tract-level) WMH volumes. Expanding on this, we evaluated the performance of voxel-based lesion-symptom mapping in predicting cognitive domain scores through a supplementary analysis. Therefore, we leveraged multivariate sparse canonical correlation analysis (SCCAN) as implemented in lesymap (https://github.com/dorianps/LESYMAP) following previous procedures.[4,5] We chose SCCAN as validation analyses indicate superior accuracy over mass univariate approaches.[4] For computational feasibility, voxels with minimal lesion coverage were excluded (fewer than 1% of patients). The model was corrected for lesion size. Cognitive domain scores were predicted out of sample via SCCAN based on voxel-level WMH segmentations in a 10-fold cross-validation using caret (v. 6.0-93, https://github.com/topepo/caret) – i.e., the voxel-level coefficients were obtained in a training set and cognitive domain scores were predicted based on applying these coefficients to unseen test data with the *lesymap.predict* function. For each fold, the pearson correlation between actual and predicted cognitive domain

scores was computed. In addition, voxel-level lesion behavior maps were obtained by averaging resulting coefficient maps across the 10 cross-validation folds.

References

1. Maillard P, Fletcher E, Harvey D, et al. White Matter Hyperintensity Penumbra. *Stroke*. 2011;42(7):1917-1922. doi:10.1161/STROKEAHA.110.609768

2. Maillard P, Fletcher E, Lockhart SN, et al. White Matter Hyperintensities and their Penumbra Lie Along a Continuum of Injury In The Aging Brain. *Stroke*. 2014;45(6):1721-1726. doi:10.1161/STROKEAHA.113.004084

3. Mayer C, Nägele FL, Petersen M, et al. Free-water diffusion MRI detects structural alterations surrounding white matter hyperintensities in the early stage of cerebral small vessel disease. *J Cereb Blood Flow Metab*. 2022;42(9):1707-1718. doi:10.1177/0271678X221093579

4. Pustina D, Avants B, Faseyitan OK, Medaglia JD, Coslett HB. Improved accuracy of lesion to symptom mapping with multivariate sparse canonical correlations. *Neuropsychologia*. 2018;115:154-166. doi:10.1016/j.neuropsychologia.2017.08.027

5. Bowren M, Adolphs R, Bruss J, et al. Multivariate Lesion-Behavior Mapping of General Cognitive Ability and Its Psychometric Constituents. *J Neurosci*. 2020;40(46):8924-8937. doi:10.1523/JNEUROSCI.1415-20.2020

Results

Figure S3 – White matter hyperintensity distribution

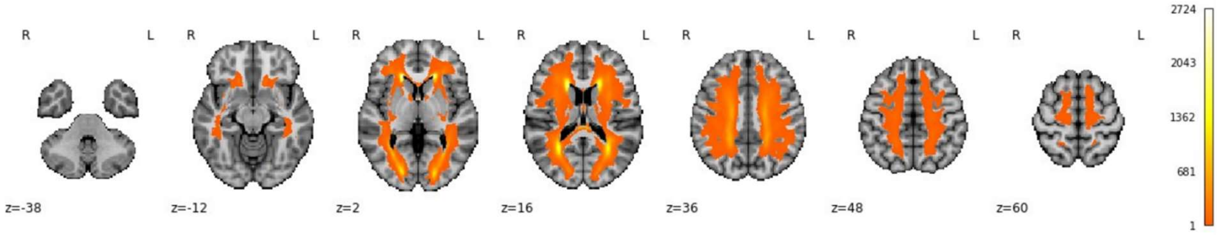

Heatmap indicating the frequency of white matter hyperintensities across the analysis sample.

Figure S4 – Predictive modeling analysis with explained variance (R^2, coefficient of determination) scoring

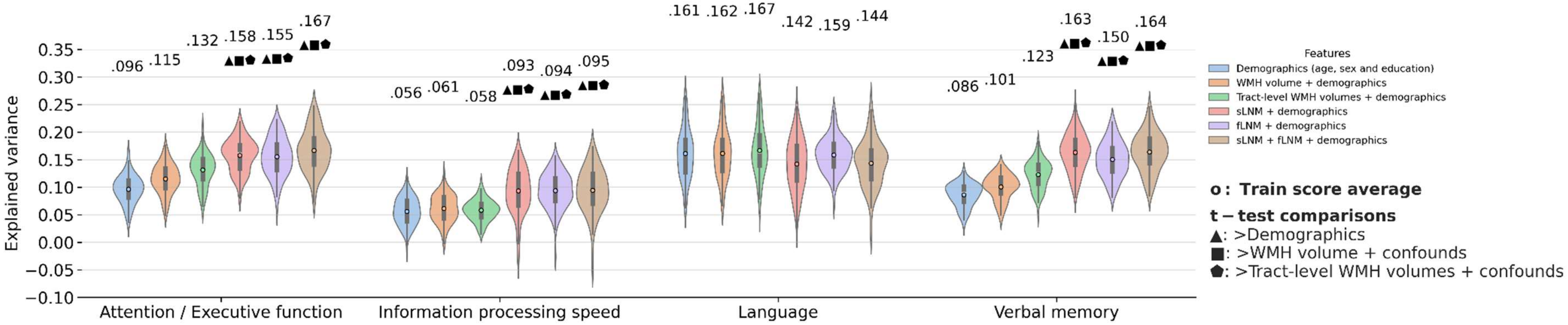

Violin plots illustrate prediction outcomes across cognitive domains. Each violin displays the distribution of explained variance of cognitive domain scores (10-fold cross-validation x 10 repeats = 100 folds → 100 Pearson correlations) for a model informed by a different feature set. The higher the explained variance, the higher the prediction performance. Blue: confounds (age, sex and education); orange: total WMH volume + confounds; green: tract-level WMH volumes + confounds; red: sLNM scores + confounds; purple: fLNM scores + confounds; brown: sLNM scores + fLNM scores + confounds. The average explained variance is indicated above each violin, with colored dots showing training score averages. Geometric symbols denote t-test results comparing LNM-based models against confound- and WMH volume-based models: ▲ indicates higher explained variance than confounds, ■ than WMH volume + confounds, ⬟ than tract-level WMH volume + confounds. Of note, a negative explained variance is possible using sum-of-squares formulation. A negative value indicates that the optimized model fits the data worse than a horizontal line representing the mean of the target variable. *Abbreviations*: fLNM = functional lesion network mapping, sLNM = structural lesion network mapping, WMH = white matter hyperintensities of presumed vascular origin.

Table S5 – Predictive modeling analysis results – Average negative mean squared error

	Attention / executive function	Information processing speed	Language	Verbal memory
Confounds (age, sex education)	-1.06219	-2.43104	-2.91271	-1.51663
WMH volume + confounds	-1.03992	-2.41763	-2.9114	-1.49302
Tract-level WMH volumes + con-founds	-1.02051	-2.42529	-2.8931	-1.45587
sLNM + confounds	-0.98846	-2.33568	-2.98366	-1.38662
fLNM + confounds	-0.99139	-2.33465	-2.92767	-1.40741
sLNM + fLNM + confounds	-0.97774	-2.33223	-2.97935	-1.38486

Figure S6 – Region of interest-level averages of lesion network mapping scores

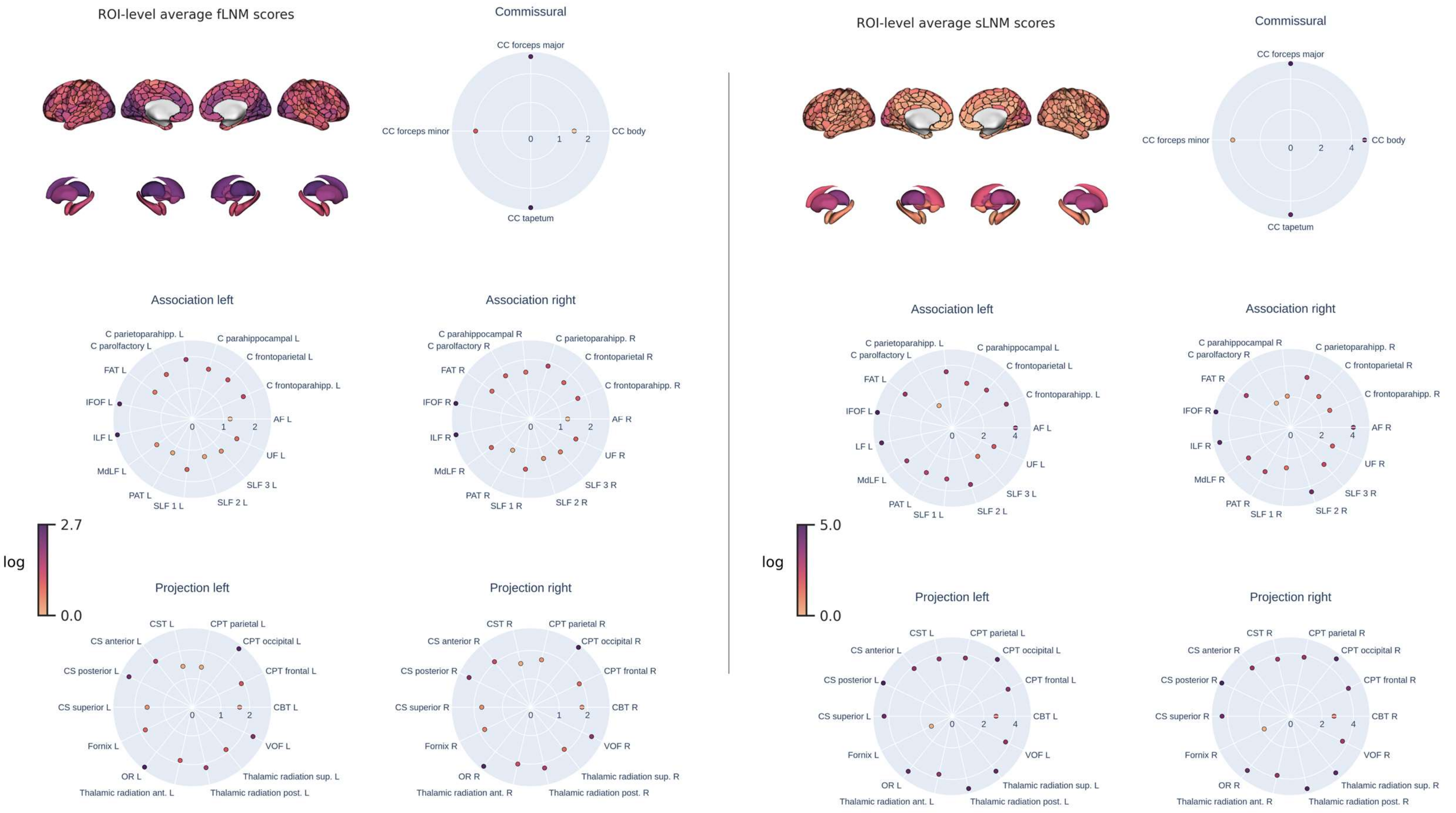

Region of interest-level average functional and structural lesion network mapping scores. Average lesion network mapping scores of gray matter regions of interest are mapped on the cortical surface and subcortical regions. For the white matter tracts, average lesion network mapping scores are displayed in radar plots. Radar plots display white matter tracts in alphabetical order starting at the 3 o'clock position.

Figure S7 – Tract-level functional lesion network mapping

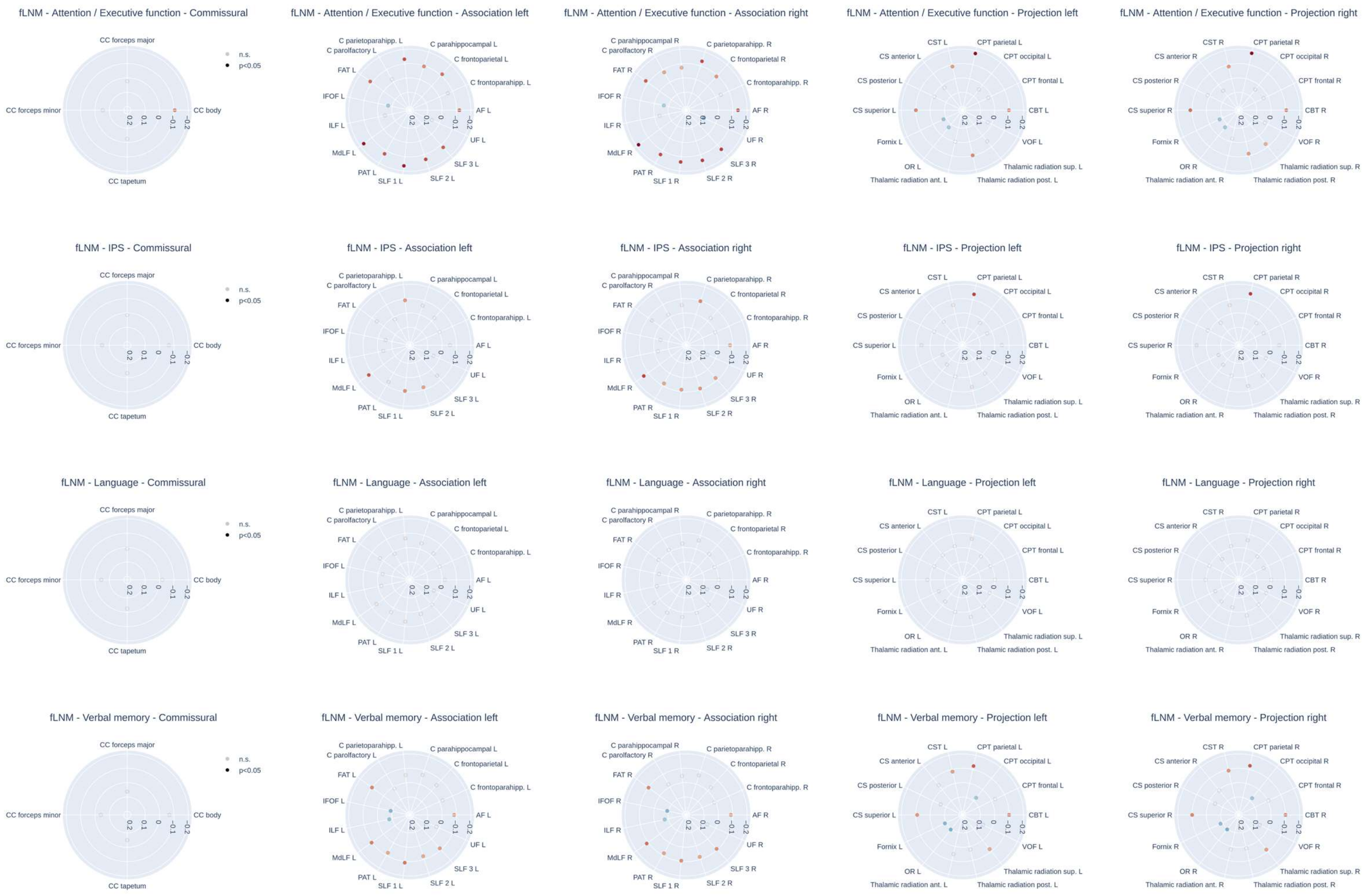

Radar plots display tract-level β coefficients from inferential statistics indicating the relationship between regional functional lesion network mapping scores and cognitive domain scores. This plot shows the associations for all tracts while in the main manuscript only the top 10 effects per combination of LNM modality and cognitive domain are featured. In contrast to the main manuscript, tracts are displayed in alphabetical order starting at the 3 o'clock position in the counterclockwise position. Red dots indicate a negative association (higher LNM score – lower cognitive domain score) and blue dots indicate a positive association (higher LNM score – higher cognitive domain score). Faintly colored dots indicate non-significant associations. Tracts with a significant association are displayed below the radar plots in alphabetical order. For paired tracts only left side examples are visualized.

Figure S8 – Tract-level structural lesion network mapping

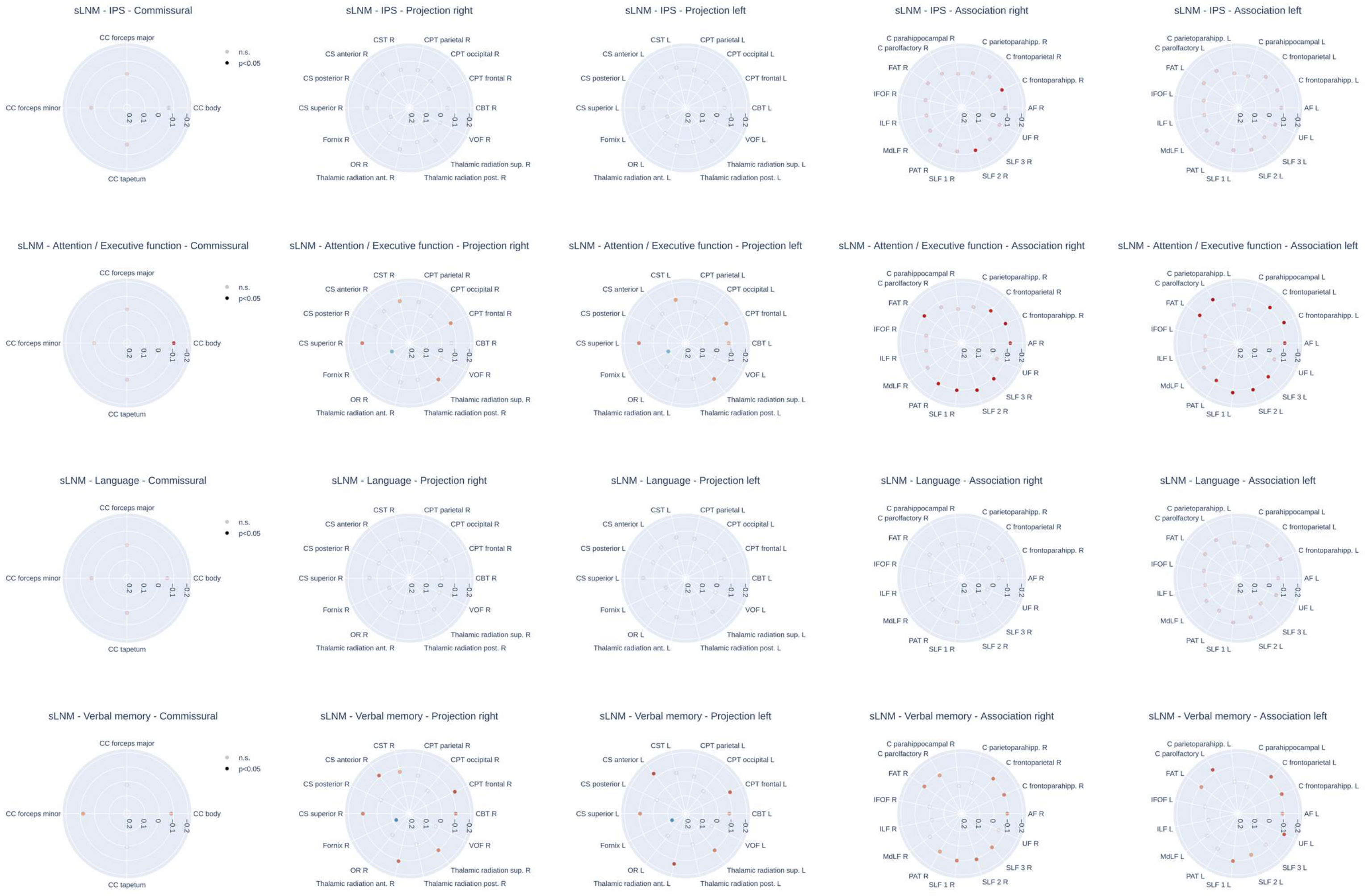

Radial plots display tract-level β coefficients from inferential statistics indicating the relationship between regional structural lesion network mapping scores and cognitive domain scores. This plot shows the associations for all tracts while in the main manuscript only the top 10 effects per combination of LNM modality and cognitive domain are featured. In contrast to the main manuscript, tracts are displayed in alphabetical order starting at the 3 o'clock position in the counterclockwise position. Red dots indicate a negative association (higher LNM score – lower cognitive domain score) and blue dots indicate a positive association (higher LNM score – higher cognitive domain score). Faintly colored dots indicate non-significant associations. Tracts with a significant association are displayed below the radar plots in alphabetical order. For paired tracts only left side examples are visualized.

Figure S9 – Spatial correlations of region of interest-level β coefficients

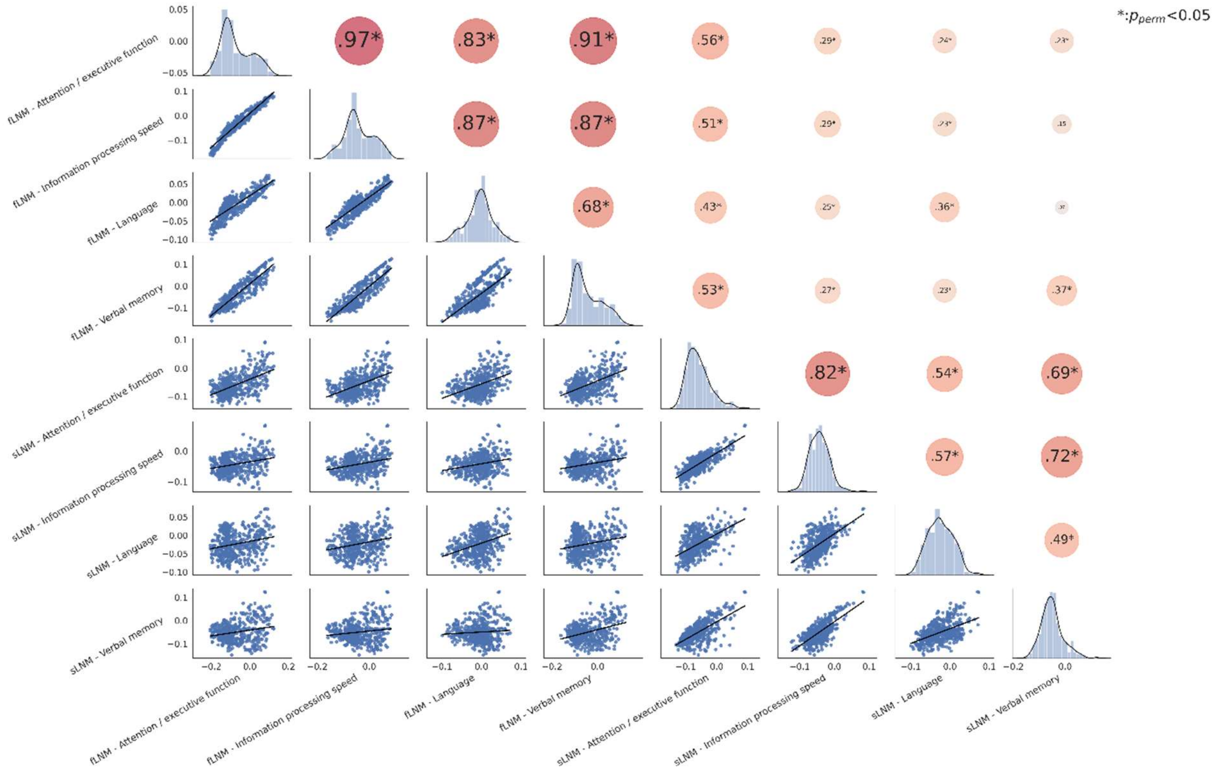

Spatial correlation matrix of all ROI-level effect maps (β). To investigate the spatial correspondence between effect maps of the ROI-level analysis, we performed Spearman correlations of each pair of maps. The upper triangle of the matrix displays spearman correlations with dot size and color representing the orientation and magnitude of the correlation coefficients. Asterisks highlight significant correlations after permutation testing and false discovery rate correction. The diagonal shows kernel density plots. The lower triangle illustrates the linear relationships via regression plots. Each dot of the regression plot corresponds with a ROI. *Abbreviations*: fLNM = functional lesion network mapping, p_{perm} = p-value obtained via comparison of empirical Spearman correlation to permutation-based null distribution, ROI = region of interest, r_{sp} = Spearman correlation, sLNM = structural lesion network mapping.

Interpretation – The β-coefficient maps representing spatial effect patterns, showed relevant overlap with 26 of 28 effect pattern pairs being significantly correlated.

Figure S10 – Sensitivity analysis: Predictive modeling analysis

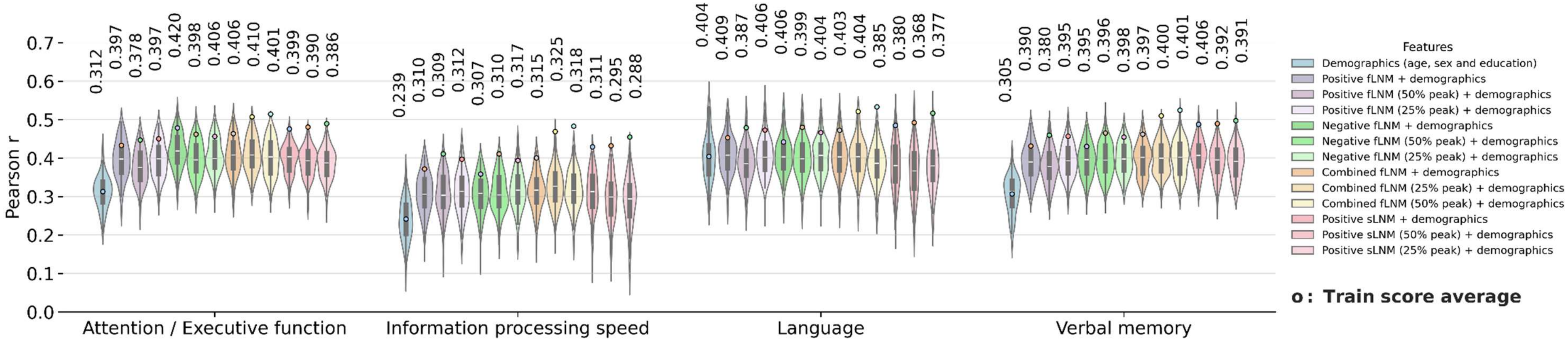

This plot corresponds with Figure 2 of the main manuscript but displays model performances informed by negative fLNM scores as well as LNM scores computed via different thresholding schemes alongside original LNM-informed models. Negative fLNM scores were obtained by only considering negative Pearson correlation coefficients within the WMH mask. Thresholding was performed by averaging only the highest 25% (25% peak) and highest 50% (50% peak) of intensity values of the ROI-level connectivity map in the WMH mask. For the negative fLNM scores, the lowest 25% and 50% voxel intensity values were averaged instead. Combined fLNM indicates models informed by positive and negative fLNM scores. *Abbreviations*: fLNM = functional lesion network mapping, sLNM = structural lesion network mapping, WMH = white matter hyperintensities of presumed vascular origin.

Interpretation – Predictive modeling results were stable when using negative fLNM scores (based on anti-correlations in resting-state fMRI measures) and when including a 25% or 50% thresholding step.

Figure S11 – Sensitivity analysis: Inferential statistics results of cortical and subcortical gray matter based on negative functional lesion network mapping scores

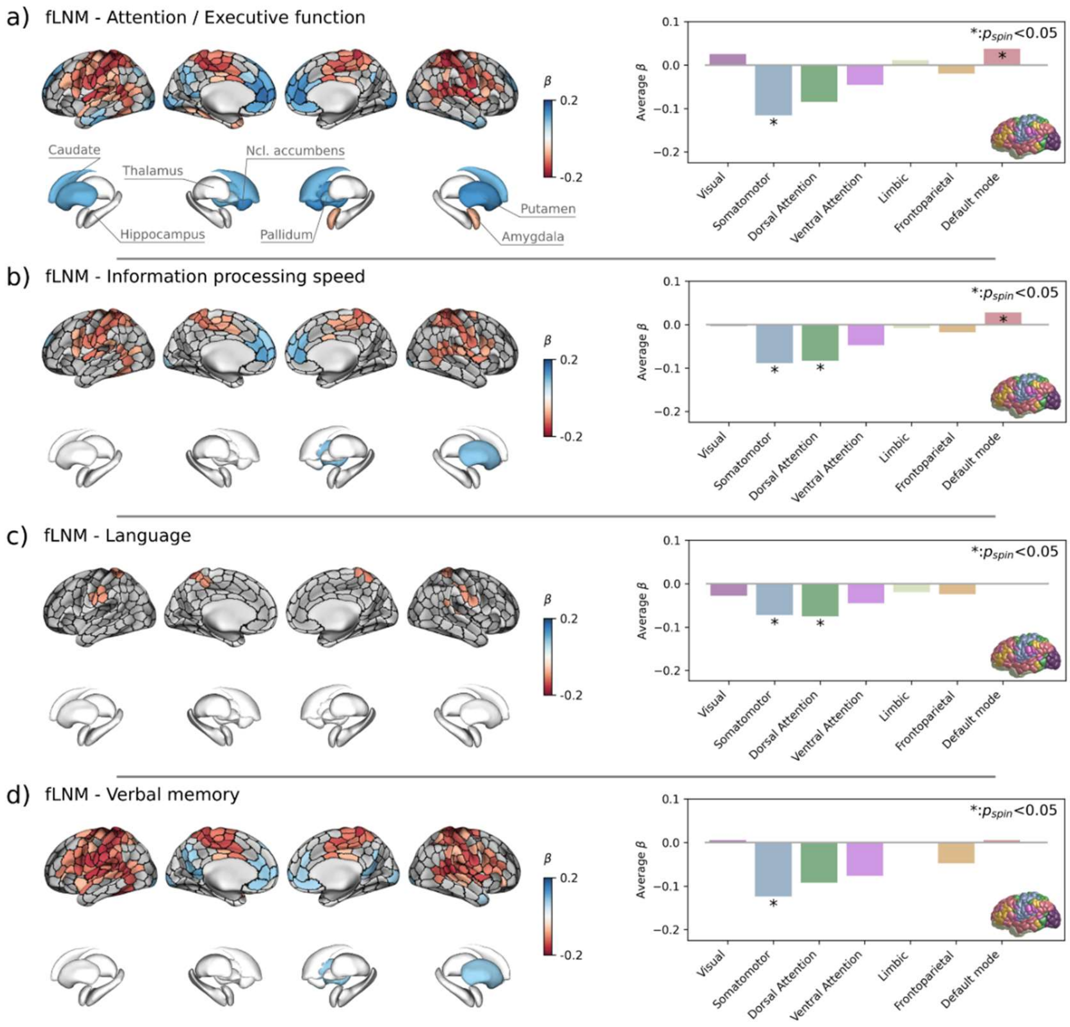

This plot corresponds with Figure 3 a) – d) of the main manuscript but in contrast displays regional associations of fLNM scores based on anticorrelations. Left: ROIs that were significantly associated with cognitive domain scores after family wise error-correction are highlighted by colors encoding β-coefficients from general linear models: a negative β (red) denotes that a higher regional LNM score, i.e., higher WMH connectivity, is associated to a lower cognitive domain performance; a positive β (blue) indicates that a higher regional LNM score is linked to a higher cognitive domain performance. Right: Barplots displaying the average β in the canonical (Yeo) resting state networks. The brain on the right indicates the regional distribution of the canonical resting state networks with colors corresponding to the bars. Statistical significance was assessed using spin permutations. Each row corresponds with a different combination of lesion network mapping modality and cognitive domain: a) fLNM – attention / executive function, b) fLNM – information processing speed, c) fLNM – language, d) fLNM – verbal memory. *Abbreviations*: fLNM = functional lesion network mapping, p_{spin} = p-value derived from spin permutations, ROIs = regions of interest, sLNM = structural lesion network mapping.

Interpretation - Stronger anticorrelation between the default mode network and WMH – reflected by more negative fLNM scores – correlates with reduced attention, executive function, and processing speed.

Figure S12 – Sensitivity analysis: Inferential statistics of white matter tracts based on negative functional lesion network mapping scores

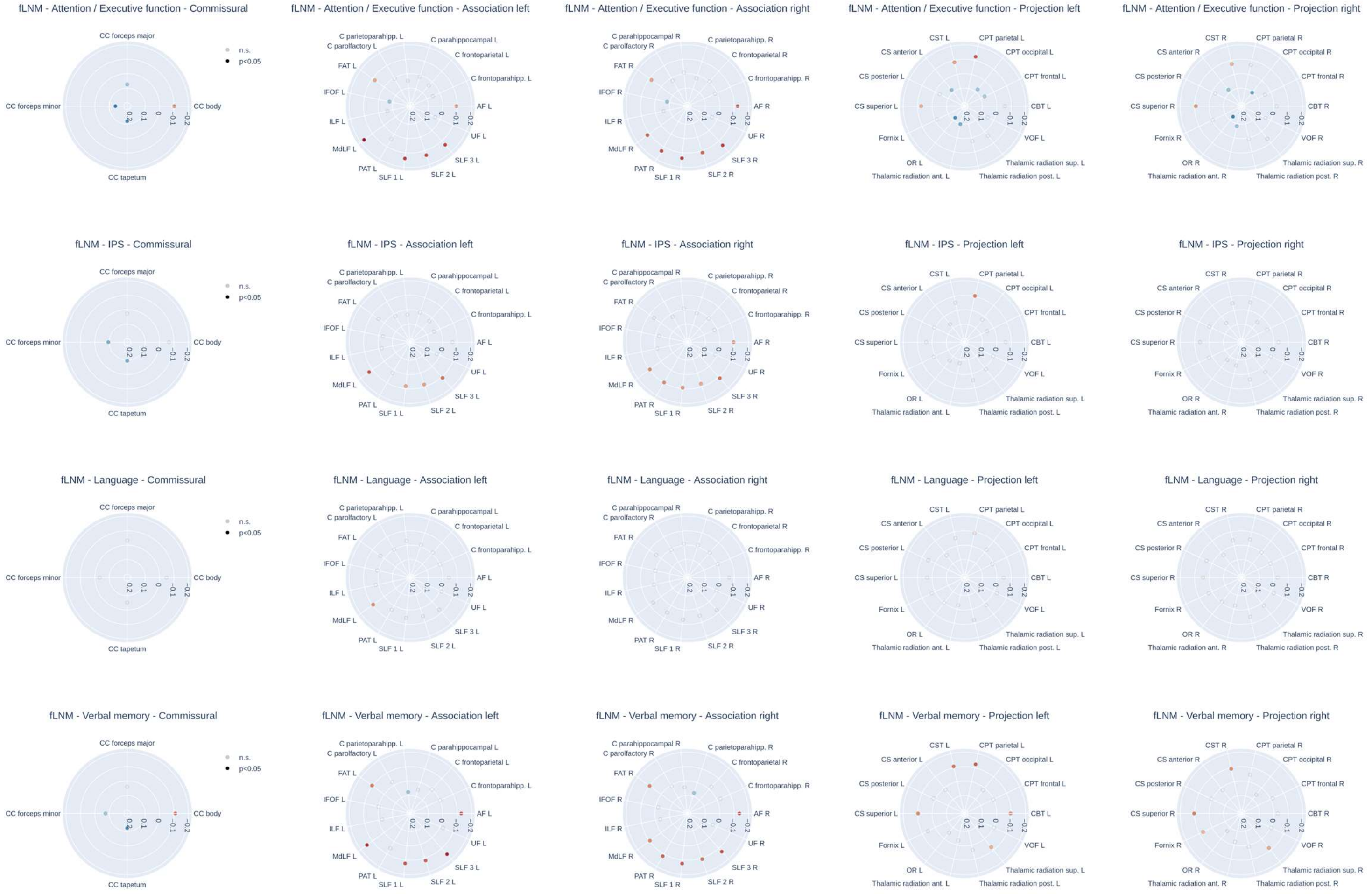

This plot corresponds with Figure 4 of the main manuscript but in contrast displays regional associations of fLNM scores based on anticorrelations. Tract abbreviations: Commissural tracts – CC = corpus callosum; Association tracts - AF = arcuate fascicle, C = cingulate, FAT = frontal aslant tract, IFOF = inferior fronto-occipital fasciculus, ILF = inferior longitudinal fasciculus, MdLF = middle longitudinal fasciculus, PAT = posterior aslant tract, SLF = superior longitudinal fasciculus, UF = uncinate fasciculus; Projection tracts – CBT = corticobulbar tract, CPT = corticopontine tract, CS = corticostriatal pathway, CST = corticospinal tract, CT = corticothalamic pathway, FPT = frontopontine tract, F = fornix, OPT = occipitopontine tract, OR = optic radiation, VOF = Vertical occipital fasciculus. *Abbreviations*: fLNM = functional lesion network mapping, n.s. = non-significant, p = p-value, sLNM = structural lesion network mapping.

Figure S13 – Sensitivity analysis: Predictive modeling on LNM scores based on different Schaefer Atlas resolutions

Schaefer100x7

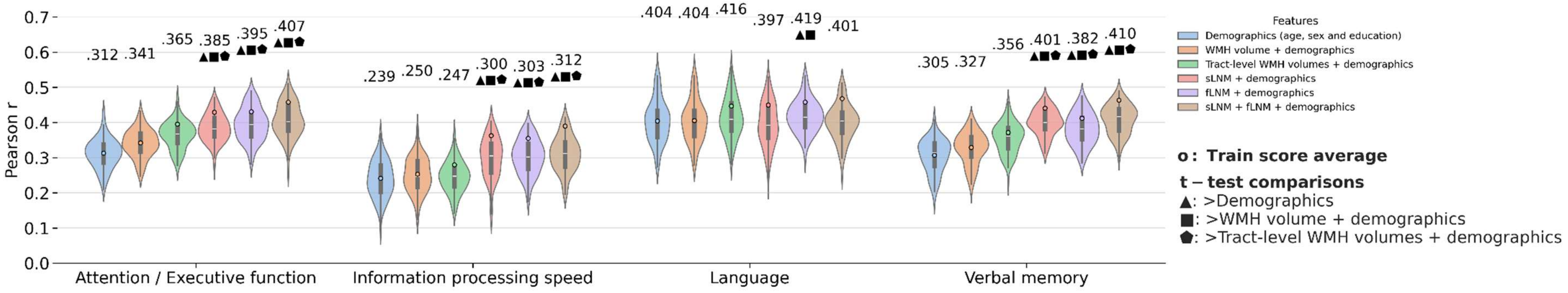

Schaefer200x7

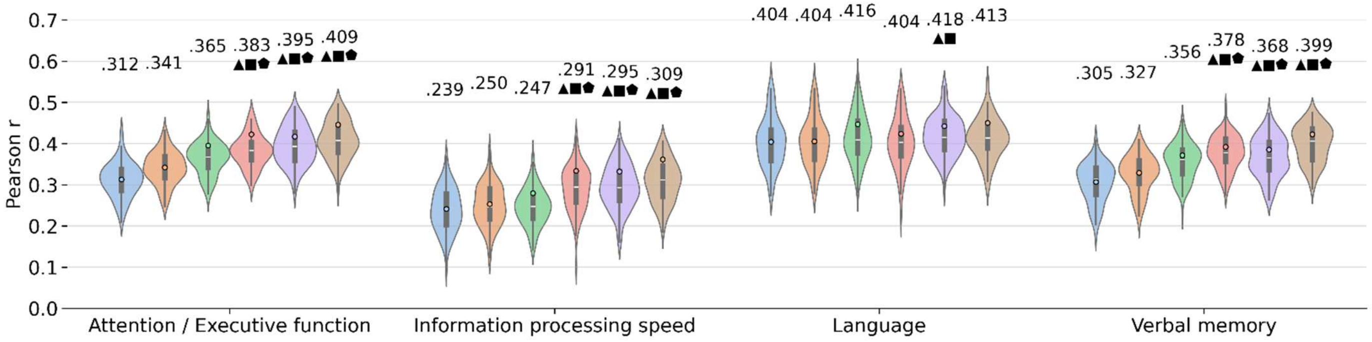

Violin plots illustrate out-of-sample prediction performance of cognitive domain scores based on demographics, WMH volume measures and LNM scores based on different Schaefer Atlas resolutions. *Abbreviations*: fLNM = functional lesion network mapping, sLNM = structural lesion network mapping, WMH = white matter hyperintensities of presumed vascular origin.

Interpretation – Models informed by LNM scores derived from lower Schaefer Atlas resolutions showed robust performance suggesting that the results of the main analysis are likely not biased by the choice of atlas resolution.

Figure S14 – Sensitivity analysis: Prediction of language function based on LNM scores of left-hemispheric WMH

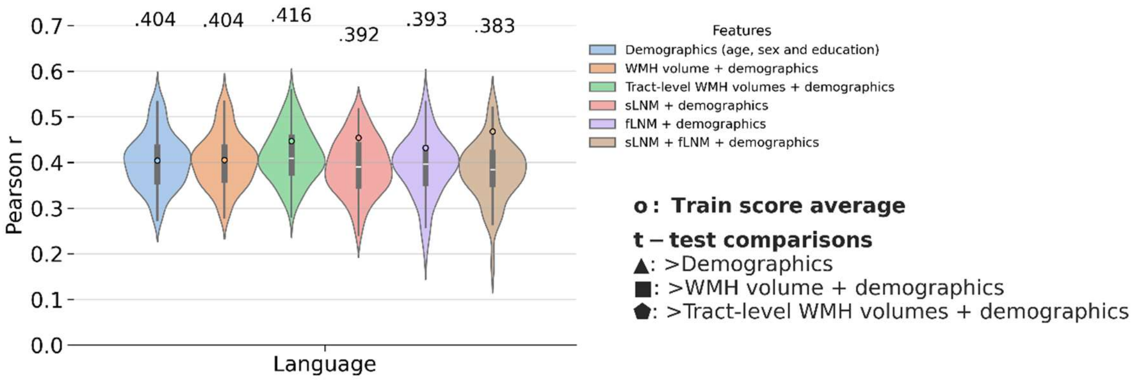

Violin plots illustrate out-of-sample prediction performance of language function based on demographics, WMH volume measures and LNM scores based only on left-hemispheric WMH. *Abbreviations*: fLNM = functional lesion network mapping, sLNM = structural lesion network mapping, WMH = white matter hyperintensities of presumed vascular origin.

Interpretation – Models informed by LNM scores based on left-hemispheric WMH remained non-superior to models informed by demographics and WMH volume measures. Thus, our results do not indicate a confounding effect of disconnection originating from the right hemisphere in language functions.

Figure S15 – Predictive modeling based on WMH penumbra-informed LNM

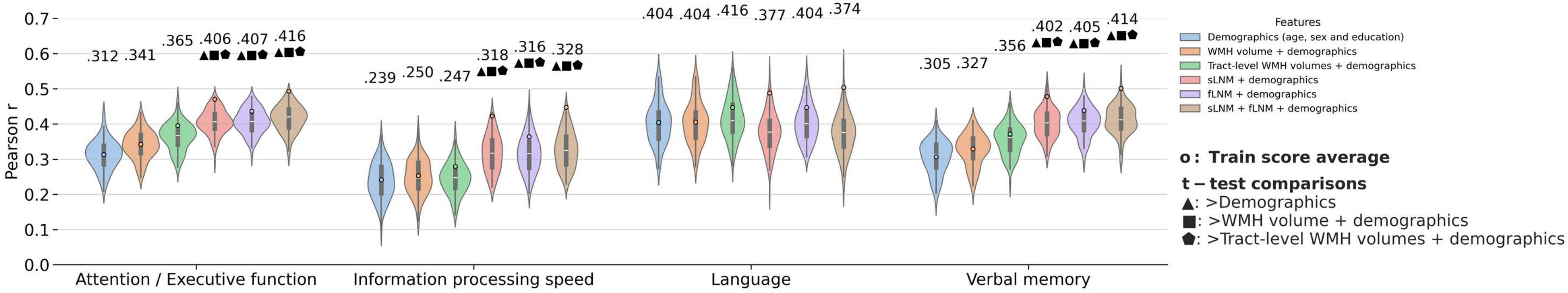

Violin plots illustrate out-of-sample prediction performance of cognitive domain scores based on demographics, WMH volume measures and LNM scores based on WMH masks three-dimensionally dilated by 2mm to include the WMH penumbra. *Abbreviations*: fLNM = functional lesion network mapping, sLNM = structural lesion network mapping, WMH = white matter hyperintensities of presumed vascular origin.

Interpretation - Including LNM scores based on both WMH and adjacent normal-appearing white matter – the so-called WMH penumbra – slightly improved predictive performance of the LNM models. This suggests that white matter abnormalities beyond visible lesions contribute to cognitive variance in memory clinic patients, reflecting the notion that small vessel pathology is widespread and diffuse. Future analyses leveraging CSVD imaging features beyond WMH should expand on this finding.

Figure S16 – Structure-function correlations of regional lesion network mapping scores

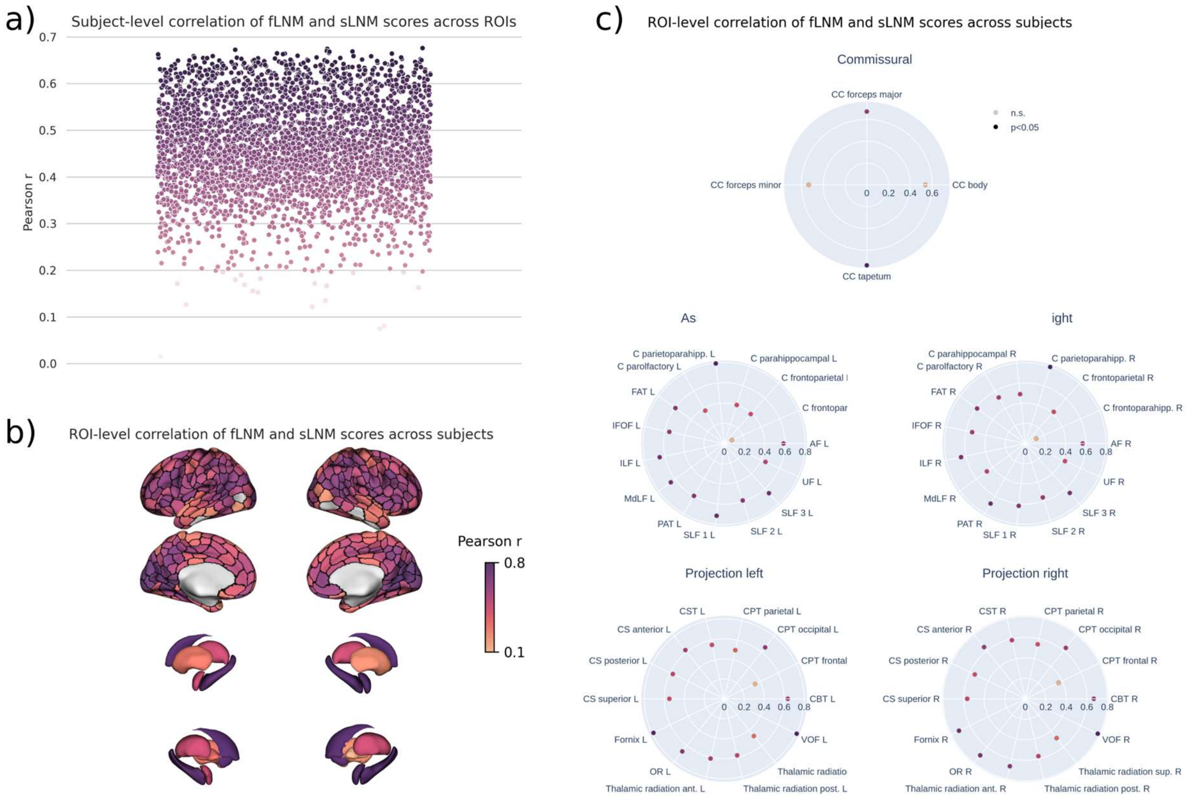

a) Swarmplot displaying the Pearson correlation of fLNM and sLNM scores across ROIs per subject. Each dot represents a subject and is colored by the Pearson correlation. b) and c) Pearson correlation of fLNM and sLNM scores across subjects per ROI. *Abbreviations*: fLNM = functional lesion network mapping, ROI = region of interest, sLNM = structural lesion network mapping.

Figure S17 – Voxel-level lesion network maps

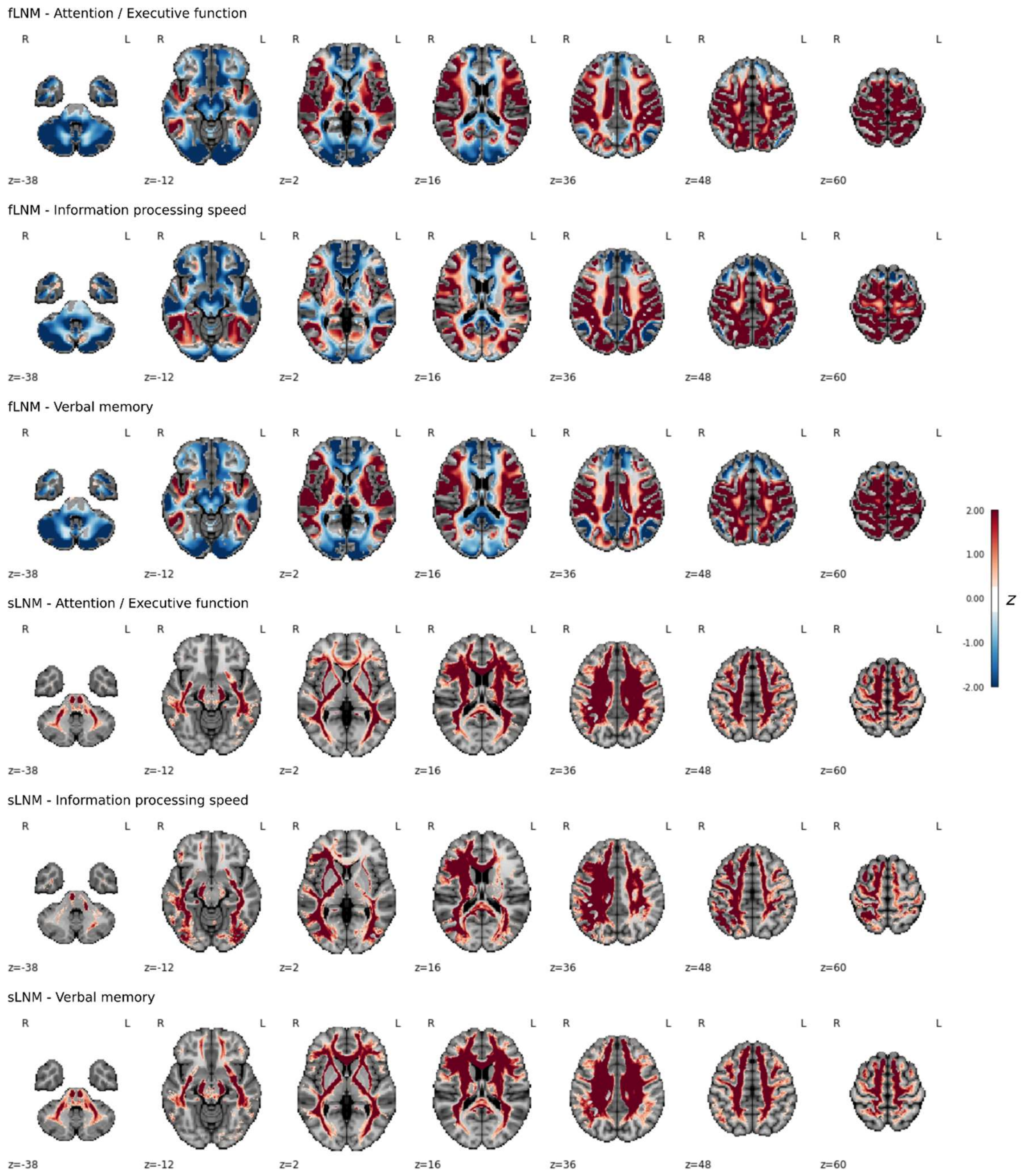

Voxel-level lesion network maps indicate the connectivity to regions of interest that significantly contribute to a cognitive domain. Each row corresponds to a different combination of lesion network mapping modalities (functional and structural) and cognitive domain scores. For the functional lesion network maps, positive z-scores indicate a positive Pearson correlation with the resting-state BOLD signal of the significantly associated ROIs. Negative z-scores indicate anticorrelated voxels and are highlighted in blue. For the structural lesion network maps, deeper red indicates that a voxel is connected by a higher amount of streamlines to significantly associated ROIs. *Abbreviations*: ROI = region of interest.

Figure S18 – Voxel-level lesion network maps scaled by the white matter hyper-intensity distribution map

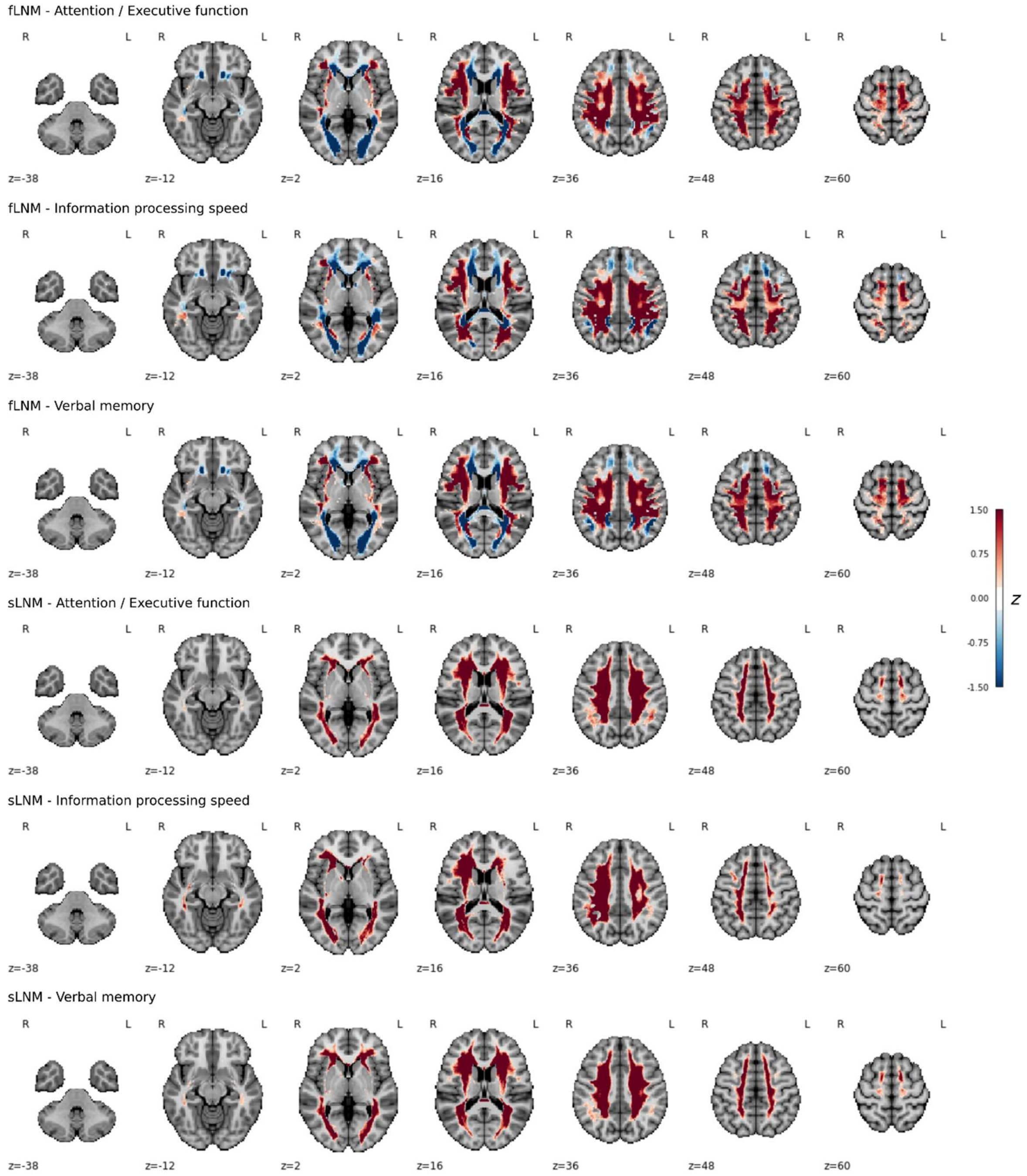

Voxel-level lesion network maps scaled by the WMH frequency map indicate the connectivity to regions of interest that significantly contribute to a cognitive domain and are likely lesioned by WMH. *Abbreviations*: ROI = region of interest, WMH = white matter hyperintensities of presumed vascular origin.

Figure S19 – Voxel-level lesion-symptom maps

Attention / executive function

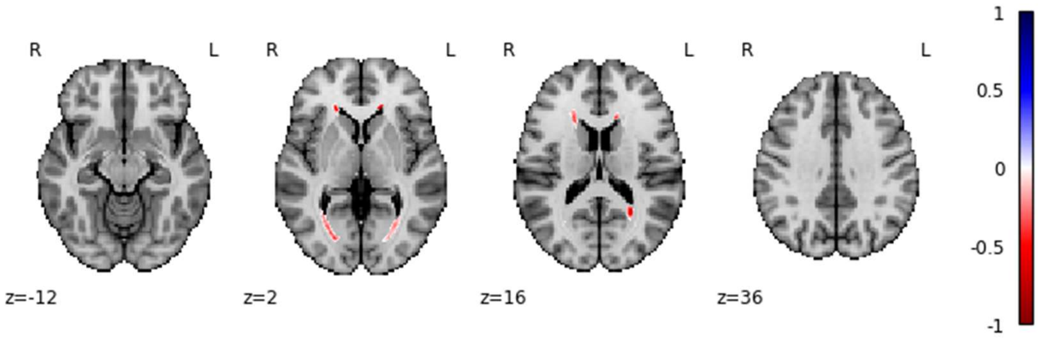

Information processing speed

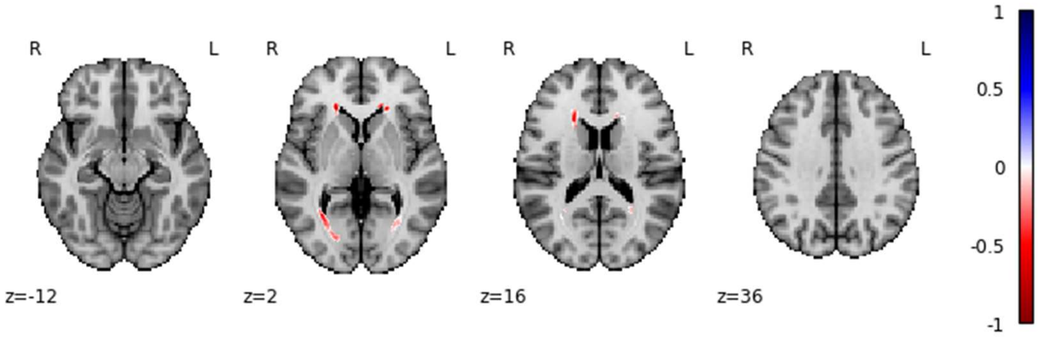

Language

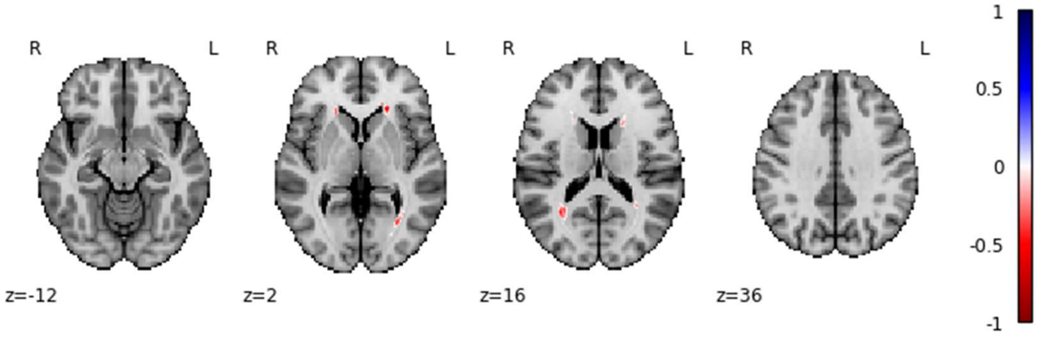

Verbal memory

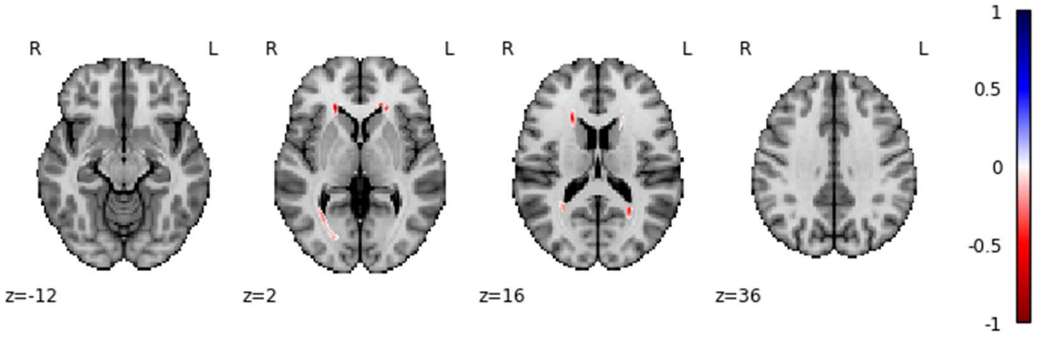

Voxel-level lesion-symptom maps. Voxel-based lesion-symptom mapping was performed employing sparse canonical correlation analysis (SCCAN) was performed in a 10-fold cross-validation and the figure displays lesion-symptom maps averaged across folds. Regions identified through SCCAN where lesion occurrence was significantly linked to cognitive domain performance are highlighted by colors encoding coefficients. Each row corresponds with a different cognitive domain score. Significant associations between lesion occurrence and lower cognitive performance were identified in bilateral periventricular regions across all cognitive domains.

Figure S20 – Prediction performance of voxel-based lesion symptom mapping

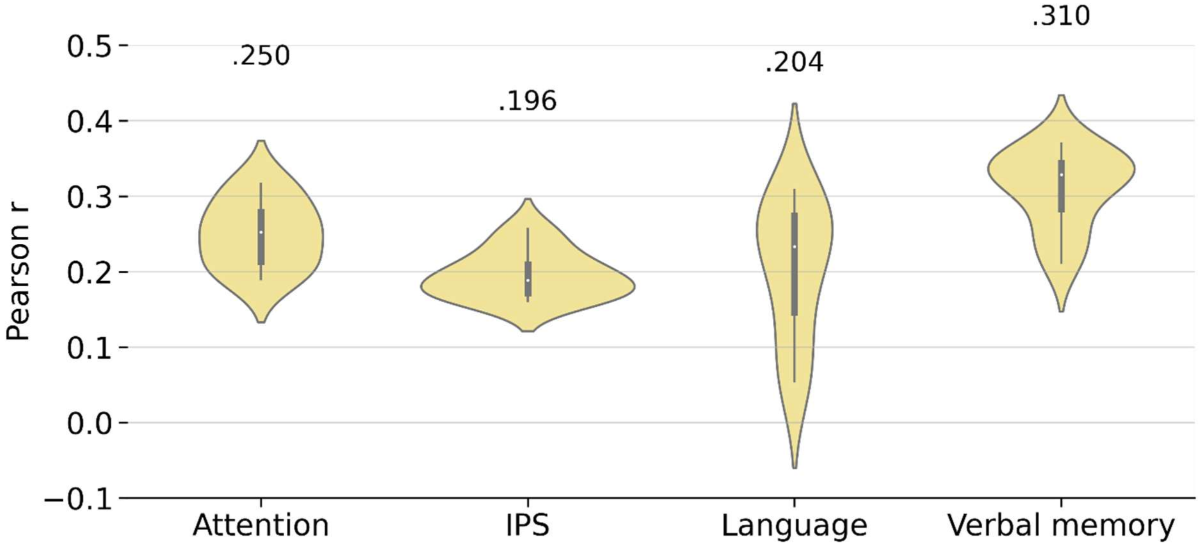

The violin plots illustrate out-of-sample prediction performance across cognitive domains based on voxel-based lesion-symptom mapping via sparse canonical correlation analysis. Each violin displays the distribution of Pearson correlation of the actual and predicted cognitive domain scores based on voxel-level WMH segmentations (10-fold cross-validation → 10 Pearson correlations). *Abbreviations*: IPS = information processing speed.

Interpretation – Compared to the predictive performance of models informed by LNM reported in the main manuscript, voxel-based lesion-symptom mapping showed lower prediction performance. This indicates that LNM captures cognitive variance beyond voxel-level lesion location.
